# Supplementary figures and images for: Disclosing proteins in the leaves of cork oak plants associated with the immune response to Phytophthora cinnamomi inoculation in the roots: A long-term proteomics approach
Source: PLoS One. 2021 Jan 22;16(1):e0245148. doi: 10.1371/journal.pone.0245148 (PMC7822296; doi:10.1371/journal.pone.0245148)

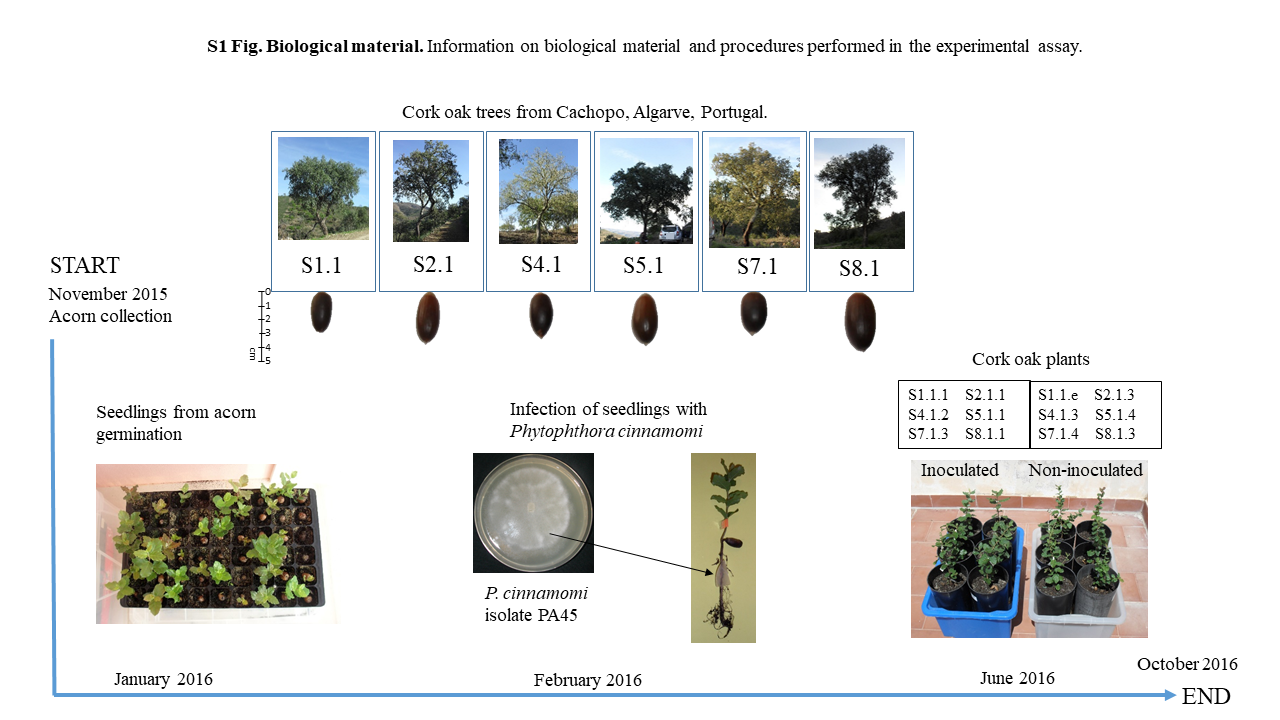

Supplement: S1 Fig — Information on biological material and procedures performed in the experimental assay. (TIF) [file pone.0245148.s001.tif]

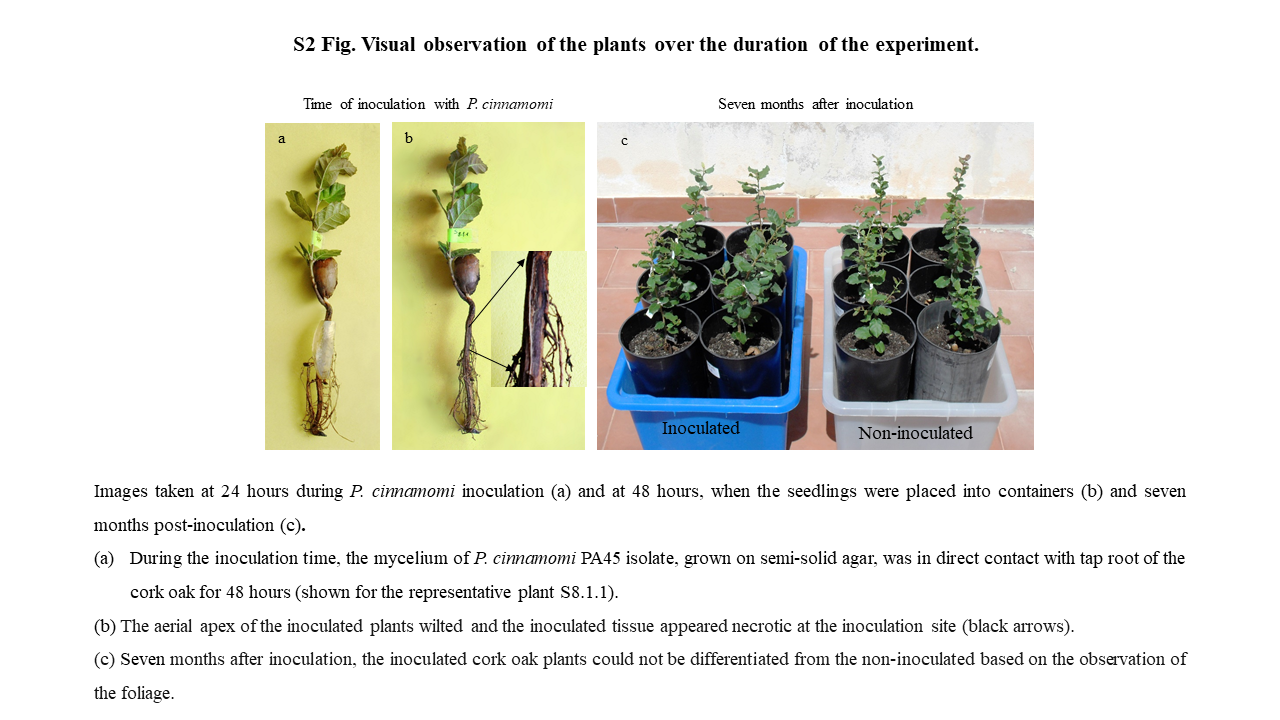

Supplement: S2 Fig — (TIF) [file pone.0245148.s002.tif]
