# Supplementary material for: Disclosing proteins in the leaves of cork oak plants associated with the immune response to Phytophthora cinnamomi inoculation in the roots: A long-term proteomics approach
Source: PLoS One. 2021 Jan 22;16(1):e0245148. doi: 10.1371/journal.pone.0245148 (PMC7822296; doi:10.1371/journal.pone.0245148)
Supplement: S1 Table — Plant labels and GPS references for location of cork oak parental trees. (PDF) [file pone.0245148.s003.pdf]

Table S1. References for cork oak plants.

Parental cork oak trees[a]	GPS references	Control plants (C)[a]	*P. cinnamomi* inoculated plants (I)[a]
S1.1[b]	37° 19.12079' N<br>7° 49.21280' W	S1.1.e[b]	S1.1.1[b]
S2.1	37° 18.40576' N<br>7° 48.97250' W	S2.1.3	S2.1.1
S4.1	37° 19.40323' N<br>7° 47.41679' W	S4.1.3	S4.1.2
S5.1	37° 19.12010' N<br>7° 47.51810' W	S5.1.4	S5.1.1
S7.1	37° 19.11278' N<br>7° 48.10301' W	S7.1.4	S7.1.3
S8.1	37° 17.52274' N<br>7° 46.44931' W	S8.1.3	S8.1.1

[a]The plants used in the experimental assay come from 6 parental cork oak trees, appearing in pairs, one in the control group (C) and the other in the inoculated group (I).

[b]Example: Cork oak tree S1.1 produced the acorns that gave rise to the plants S1.1.e (C) and S1.1.1 (I).
