# Supplementary material for: Disclosing proteins in the leaves of cork oak plants associated with the immune response to Phytophthora cinnamomi inoculation in the roots: A long-term proteomics approach
Source: PLoS One. 2021 Jan 22;16(1):e0245148. doi: 10.1371/journal.pone.0245148 (PMC7822296; doi:10.1371/journal.pone.0245148)
Supplement: S4 Table — (PDF) [file pone.0245148.s006.pdf]

Significantly enriched (FDR<0.05) GO Biological process terms and groups in the list of 80 differential proteins (p<0.05 or 0.5>FC>2 between Control and Inoculated)								
GO_ID	GO_Term	Source	Term FDR	Group FDR	Enrichment score	GO groups	% Associated proteins	Number of proteins
**GO:0072524**	**pyridine-containing compound metabolic process**	**BP**	**1.85E-04**	**1.29E-04**	**12.9**	**4**	**4.24**	**5.00**
GO:0019362	pyridine nucleotide metabolic process	BP	2.07E-04	1.29E-04	12.9	4	4.50	5.00
GO:0006733	oxidoreduction coenzyme metabolic process	BP	2.09E-04	1.29E-04	12.9	4	4.00	5.00
GO:0046496	nicotinamide nucleotide metabolic process	BP	2.64E-04	1.29E-04	12.9	4	4.55	5.00
GO:0006739	NADP metabolic process	BP	5.49E-04	1.29E-04	12.9	4	8.33	3.00
GO:0006081	cellular aldehyde metabolic process	BP	2.07E-03	1.29E-04	12.9	4	4.05	3.00
**GO:0046365**	**monosaccharide catabolic process**	**BP**	**3.34E-07**	**3.53E-04**	**11.5**	**6**	**44.44**	**4.00**
GO:0019320	hexose catabolic process	BP	2.30E-05	3.53E-04	11.5	6	37.50	3.00
GO:0072524	pyridine-containing compound metabolic process	BP	1.85E-04	3.53E-04	11.5	6	4.24	5.00
GO:0005996	monosaccharide metabolic process	BP	1.96E-04	3.53E-04	11.5	6	4.35	5.00
GO:0019362	pyridine nucleotide metabolic process	BP	2.07E-04	3.53E-04	11.5	6	4.50	5.00
GO:0006733	oxidoreduction coenzyme metabolic process	BP	2.09E-04	3.53E-04	11.5	6	4.00	5.00
GO:0046496	nicotinamide nucleotide metabolic process	BP	2.64E-04	3.53E-04	11.5	6	4.55	5.00
GO:0019318	hexose metabolic process	BP	3.77E-04	3.53E-04	11.5	6	5.19	4.00
GO:0006739	NADP metabolic process	BP	5.49E-04	3.53E-04	11.5	6	8.33	3.00
GO:0006006	glucose metabolic process	BP	6.23E-04	3.53E-04	11.5	6	7.32	3.00
GO:0009135	purine nucleoside diphosphate metabolic process	BP	1.71E-03	3.53E-04	11.5	6	4.48	3.00
GO:0009185	ribonucleoside diphosphate metabolic process	BP	1.71E-03	3.53E-04	11.5	6	4.48	3.00
GO:0009179	purine ribonucleoside diphosphate metabolic process	BP	1.71E-03	3.53E-04	11.5	6	4.48	3.00
GO:0006757	ATP generation from ADP	BP	1.71E-03	3.53E-04	11.5	6	4.48	3.00
GO:0046031	ADP metabolic process	BP	1.71E-03	3.53E-04	11.5	6	4.48	3.00
GO:0006096	glycolytic process	BP	1.71E-03	3.53E-04	11.5	6	4.48	3.00
GO:0006165	nucleoside diphosphate phosphorylation	BP	2.00E-03	3.53E-04	11.5	6	4.17	3.00
GO:0009132	nucleoside diphosphate metabolic process	BP	2.06E-03	3.53E-04	11.5	6	4.00	3.00
GO:0046939	nucleotide phosphorylation	BP	2.07E-03	3.53E-04	11.5	6	4.05	3.00
**GO:0043094**	**cellular metabolic compound salvage**	**BP**	**5.65E-04**	**5.90E-04**	**10.7**	**3**	**4.21**	**4.00**
GO:0009853	photorespiration	BP	1.14E-03	5.90E-04	10.7	3	5.56	3.00
**GO:0065004**	**protein-DNA complex assembly**	**BP**	**4.08E-04**	**9.43E-04**	**10.1**	**5**	**4.94**	**4.00**
GO:0071824	protein-DNA complex subunit organization	BP	5.23E-04	9.43E-04	10.1	5	4.40	4.00
GO:0006334	nucleosome assembly	BP	6.23E-04	9.43E-04	10.1	5	7.32	3.00
GO:0031497	chromatin assembly	BP	9.81E-04	9.43E-04	10.1	5	6.12	3.00
GO:0034728	nucleosome organization	BP	1.03E-03	9.43E-04	10.1	5	5.88	3.00
GO:0006333	chromatin assembly or disassembly	BP	1.44E-03	9.43E-04	10.1	5	4.84	3.00
GO:0006323	DNA packaging	BP	1.45E-03	9.43E-04	10.1	5	4.92	3.00
**GO:0034440**	**lipid oxidation**	**BP**	**1.14E-03**	**9.53E-04**	**10.0**	**1**	**5.56**	**3.00**
**GO:0034976**	**response to endoplasmic reticulum stress**	**BP**	**1.32E-03**	**9.79E-04**	**10.0**	**2**	**5.17**	**3.00**

The leading term of each enriched group is that with the lowest term FDR (highest enrichment score), highlighted in bold and used to name the respective group.
