# Supplementary material for: Disclosing proteins in the leaves of cork oak plants associated with the immune response to Phytophthora cinnamomi inoculation in the roots: A long-term proteomics approach
Source: PLoS One. 2021 Jan 22;16(1):e0245148. doi: 10.1371/journal.pone.0245148 (PMC7822296; doi:10.1371/journal.pone.0245148)
Supplement: S5 Table — (PDF) [file pone.0245148.s007.pdf]

Significantly enriched (FDR<0.05) GO Cellular Component terms and groups in the 80 differential proteins (p<0.05 or 0.5>FC>2 between Control and Inoculated)								
GO_ID	GO_Term	Source	Term FDR	Group FDR	Enrichement score	GO groups	% Associated proteins	Number of proteins
**GO:0044445**	**Cytosolic part**	**CC**	**4.83E-27**	**1.96E-25**	**82.1**	**4**	**7.20**	**25.00**
GO:0022626	Cytosolic ribosome	CC	1.02E-26	1.96E-25	82.1	4	7.57	24.00
GO:0005840	Ribosome	CC	1.42E-24	1.96E-25	82.1	4	5.49	25.00
GO:0044391	Ribosomal subunit	CC	2.01E-20	1.96E-25	82.1	4	6.83	19.00
GO:0022627	Cytosolic small ribosomal subunit	CC	2.85E-12	1.96E-25	82.1	4	9.17	10.00
GO:0015935	Small ribosomal subunit	CC	7.46E-12	1.96E-25	82.1	4	8.20	10.00
GO:0022625	Cytosolic large ribosomal subunit	CC	3.10E-10	1.96E-25	82.1	4	7.09	9.00
GO:0015934	Large ribosomal subunit	CC	1.61E-09	1.96E-25	82.1	4	5.81	9.00
**GO:0010319**	**Stromule**	**CC**	**1.17E-07**	**1.41E-07**	**22.8**	**1**	**13.89**	**5.00**
**GO:0000786**	**Nucleosome**	**CC**	**5.74E-07**	**3.60E-05**	**14.8**	**2**	**10.00**	**5.00**
GO:0000785	Chromatin	CC	3.38E-05	3.60E-05	14.8	2	4.24	5.00
GO:0000790	Nuclear chromatin	CC	3.65E-05	3.60E-05	14.8	2	6.90	4.00
**GO:0009521**	**Photosystem**	**CC**	**6.51E-05**	**5.64E-05**	**14.1**	**3**	**5.71**	**4.00**
GO:0009522	Photosystem I	CC	1.64E-04	5.64E-05	14.1	3	8.57	3.00
GO:0009523	Photosystem II	CC	4.71E-04	5.64E-05	14.1	3	5.88	3.00

The leading term of each enriched group is that with the lowest term FDR (highest enrichment score), highlighted in bold and used to name the respective group.
