# Supplementary material for: Disclosing proteins in the leaves of cork oak plants associated with the immune response to Phytophthora cinnamomi inoculation in the roots: A long-term proteomics approach
Source: PLoS One. 2021 Jan 22;16(1):e0245148. doi: 10.1371/journal.pone.0245148 (PMC7822296; doi:10.1371/journal.pone.0245148)
Supplement: S6 Table — (PDF) [file pone.0245148.s008.pdf]

Significantly enriched (FDR<0.05) GO Biological process groups in the list of 80 differential proteins								
GO_ID	GO_Term	Ontology source	Term FDR	Group FDR	Enrichment score	GO groups	% Associated Proteins	Number of proteins
GO:0072524	Pyridine-containing compound metabolic process	GO_BP	1.85E-04	1.29E-04	12.9	4	4.24	5.00
GO:0046365	Monosaccharide catabolic process	GO_BP	3.34E-07	3.53E-04	11.5	6	44.44	4.00
GO:0043094	Cellular metabolic compound salvage	GO_BP	5.65E-04	5.90E-04	10.7	3	4.21	4.00
GO:0065004	Protein-DNA complex assembly	GO_BP	4.08E-04	9.43E-04	10.1	5	4.94	4.00
GO:0034440	Lipid oxidation	GO_BP	1.14E-03	9.53E-04	10.0	1	5.56	3.00
GO:0034976	Response to endoplasmic reticulum stress	GO_BP	1.32E-03	9.79E-04	10.0	2	5.17	3.00
