# Supplementary material for: Disclosing proteins in the leaves of cork oak plants associated with the immune response to Phytophthora cinnamomi inoculation in the roots: A long-term proteomics approach
Source: PLoS One. 2021 Jan 22;16(1):e0245148. doi: 10.1371/journal.pone.0245148 (PMC7822296; doi:10.1371/journal.pone.0245148)
Supplement: S7 Table — (PDF) [file pone.0245148.s009.pdf]

Significantly enriched (FDR<0.05) GO Cellular Component groups in the list of 80 differential proteins								
GO_ID	GO_Term	Ontology source	Term FDR	Group FDR	Enrichement score	GO groups	% Associated proteins	Number of proteins
GO:0044445	Cytosolic part	GO_CC	4.83E-27	1.96E-25	82.1	4	7.20	25.00
GO:0010319	Stromule	GO_CC	1.17E-07	1.41E-07	22.8	1	13.89	5.00
GO:0000786	Nucleosome	GO_CC	5.74E-07	3.60E-05	14.8	2	10.00	5.00
GO:0009521	Photosystem	GO_CC	6.51E-05	5.64E-05	14.1	3	5.71	4.00
