# Supplementary material for: Disclosing proteins in the leaves of cork oak plants associated with the immune response to Phytophthora cinnamomi inoculation in the roots: A long-term proteomics approach
Source: PLoS One. 2021 Jan 22;16(1):e0245148. doi: 10.1371/journal.pone.0245148 (PMC7822296; doi:10.1371/journal.pone.0245148)
Supplement: S1 File — Principles and detailed materials and methods. (PDF) [file pone.0245148.s010.pdf]

S1 File - Description of the SWATH-MS, principles and detailed materials and methods.

1. Introduction

In mass spectrometry-based proteomics screenings, label-free methods still rely on peptide identification through identification-dependent acquisition (IDA) experiments. However, these methods tend to be biased to the most abundant proteins and are highly affected by sample complexity and dynamic range [1], making them very useful for protein identification purposes but biased for quantitative approaches. This way, the use of data-independent acquisition (DIA) methods, where fragmentation spectra are acquired for the entire sample without any pre-selection of precursor ions, have started to be used for label-free quantitative approaches as an alternative to the limitations of IDA experiments [2, 3].

In 2012, SWATH-MS was introduced [4] and this method was particularly innovative due to the proposed data extraction methodology, with a targeted data extraction by combining parallel analysis of samples with an optimized IDA method for peptide identification followed by a DIA acquisition to be used to extract quantitative information. From the IDA method, a list (called "library") containing all the information regarding a given identified peptide (such as RT, precursor m/z and MS/MS spectra) is obtained and is further used to extract data of the specific fragment ions from all the high confidence peptides identified. Thus, instead of using the precursor intensity as performed by the other methods, in SWATH-MS the signaling intensity is used for untargeted analysis of large fractions of the proteome.

The SWATH-MS method is able to overcome many limitations of other label-free methods, because it is unbiased; it is able to have a comprehensive range of precursor ion fragmentation, being able to cover almost the entire mass range typically analyzed; and it relies on targeted data extraction [4]. All these reasons make SWATH a promising strategy for the quantitative screening of large number of proteins that has previously been applied in the field of plant biology [5-7] and recognized as a valuable tool for the comprehensive study of proteins in plants [8, 9].

2. Detailed Materials and Methods

Sample preparation

Upon arrival the samples were stored at -80˚C until further processing.

After thawing, 10 µg of each sample from each group were pooled together, creating this way two pooled samples (Pool of control samples and infected samples). These pooled sample were created for library construction.

At this point the same amount of a recombinant protein (Green fluorescent Protein and Maltose-binding periplasmic protein (malE-GFP)) was added to the volume corresponding to 50 µg of each sample and to the pools to serve as an internal standard. All the samples were boiled for 5 minutes and acrylamide was added as an alkylating agent.

In-gel digestion and liquid chromatography coupled to tandem mass spectrometry (LC-MS/MS)

All samples and pools were then loaded into a precast gel (4–20% Mini-Protean® TGX™ Gel, Bio-Rad), as well as the pooled samples, and the SDS-PAGE was partially run for 20 minutes at 110V [10]. After SDS-PAGE, proteins were stained with Colloidal Coomassie Blue as previously described [11].

The lanes were sliced into 3 fractions with the help of a scalpel, and after the excision of the gel bands each one was sliced into smaller pieces. The gel pieces were destained using a 50 mM ammonium bicarbonate solution with 30% acetonitrile (ACN) followed by a washing step with water (each step was performed in a thermomixer (Eppendorf) at 1050 rpm for 15 min). The gel pieces were dehydrated on Concentrador Plus/Vacufuge® Plus (Eppendorf). To each gel band 75 µL of trypsin (0.01 µg/µL solution in 10 mM ammonium bicarbonate) were added to the dried gel bands and left for 15 min at 4°C to rehydrate the gel pieces. After this period 75 µL of 10 mM ammonium bicarbonate were added and in-gel digestion was performed overnight at room temperature in the dark. After digestion, the excess solution from gel pieces was collected to a low binding microcentrifuge tube (LoBind®, Eppendorf) and peptides were extracted from the gel pieces by sequential addition of three solutions of increasing percentage of acetonitrile (30%, 50%, and 98%) in 1% formic acid (FA). After the addition of each solution, the gel pieces were shaken in a thermomixer (Eppendorf) at 1250 rpm for 15 min and the solution was collected to the tube containing the previous fraction. The peptide mixtures were dried by rotary evaporation under vacuum (Concentrador Plus/Vacufuge® Plus, Eppendorf). The peptides from each fraction of each sample were pooled together for SWATH analysis; the peptides from the pooled samples were kept separated in the three fractions of the digestion procedure.

After digestion, all samples were subjected to solid phase extraction with C18 sorbent (OMIX tip, Agilent Technologies). The eluted peptides were evaporated and solubilised in mobile phase, aided by ultrasonication using a cup horn device (Vibra-cell 750 watt, Sonics) at 40% amplitude for 2 minutes. Samples were then centrifuged for 5 minutes at 14,100xg (minispin plus, Eppendorf) and analysed by LC-MS/MS.

The Triple TOF™ 5600 System (Sciex) was operated in two phases: information-dependent acquisition (IDA) of each fraction of the pooled samples; followed by SWATH (Sequential Windowed data independent Acquisition of the Total High-resolution Mass Spectra) acquisition of each sample. Peptide separation was performed using liquid chromatography (nanoLC Ultra 2D, Eksigent) on a ChromXP C18CL reverse phase column (300 μm × 15 cm, 3 μm, 120Å, Eksigent) at 5 μL/min with a 45 min linear gradient from 5% to 30% acetonitrile in 0.1% FA, and the peptides were eluted into the mass spectrometer using an electrospray ionization source (DuoSpray™ Source, Sciex).

Information dependent acquisition (IDA) experiments were performed by analysing 10 μL of each fraction of the pooled samples. The mass spectrometer was set for IDA scanning full spectra (350-1250 m/z) for 250 ms, followed by up to 100 MS/MS scans (100–1500 m/z from a dynamic accumulation time – minimum 30 ms for precursor above the intensity threshold of 1000 counts per second (cps) – in order to maintain a cycle time of 3.3 s). Candidate ions with a charge state between +2 and +5 and counts above a minimum threshold of 10 cps were isolated for fragmentation and one MS/MS spectra was collected before adding those ions to the exclusion list for 25 seconds (mass spectrometer operated by Analyst® TF 1.7, Sciex). Rolling collision energy was used with a collision energy spread of 5.

The SWATH setup was essentially as in Gillet et al [12], with the same chromatographic conditions used for SWATH and IDA acquisitions. For SWATH-MS based experiments, the mass spectrometer was operated in a looped product ion mode. The SWATH-MS setup was designed specifically for the samples to be analysed (S1 Table 1), in order to adapt the SWATH windows to the complexity of this batch of samples. A set of 60 windows of variable width (containing 1 m/z for window overlap) was constructed covering the precursor mass range of 350-1250 m/z. A 250 ms survey scan (350-1250 m/z) was acquired at the beginning of each cycle for instrument calibration and SWATH MS/MS spectra were collected from 100–1500 m/z for 50 ms resulting in a cycle time of 3.3 s from the precursors ranging from 350 to 1250 m/z. The collision energy for each window was determined according to the calculation for a charge +2 ion centered upon the window with variable collision energy spread (CES) according with the window.

Protein identification and relative quantification

Specific library of precursor masses and fragment ions were created by combining all files from the IDA experiments, and used for subsequent SWATH processing. The libraries were obtained using ProteinPilot™ software (v5.0.1, Sciex), with the following databases: 1) the reviewed

entries for *plant* from Swiss-Prot database (accessed in April 2018); 2) the reference proteomes downloaded from Uniprot (April 2018) for *Populus trichocarpa* (UP000006729) and *Arabidopsis thaliana (*UP000006548) or 3) the predicted proteins deduced from the recently published draft genome of *Quercus suber* (downloaded from the CorkOakDB at http://corkoakdb.org/downloads in November 2020). To all databases the sequence of malE-GFP was added and acrylamide alkylated cysteines were defined as fixed modification and the gel based special focus option. An independent False Discovery Rate (FDR) analysis using the target-decoy approach provided with ProteinPilot™ software was used to assess the quality of the identifications, and positive identifications were considered when identified proteins and peptides reached a 5% local FDR. [13, 14]

Data processing was performed using SWATH™ processing plug-in for PeakView™ (v2.2, Sciex), briefly peptides were selected from the library (either *Arabidopsis thaliana* or *Quercus suber*) using the following criteria: (i) the unique peptides for a specific targeted protein were ranked by the intensity of the precursor ion from the IDA analysis as estimated by the ProteinPilot™ software, and (ii) Peptides that contained biological modifications and/or were shared between different protein entries/isoforms were excluded from selection. Up to 15 peptides were chosen per protein, and SWATH™ quantitation was attempted for all proteins in the library file that were identified below 5% local FDR from ProteinPilot™ searches. Peptide's retention time was adjusted by using the malE-GFP peptides. In SWATH™ Acquisition data, peptides are confirmed by finding and scoring peak groups, which are a set of fragment ions for the peptide. Up to 5 target fragment ions were automatically selected and the peak groups were scored following the criteria described in Lambert et al [15]. Peak group confidence threshold was determined based on a FDR analysis using the target-decoy approach and 1% extraction FDR threshold was used for all the analyses. Peptides that met the 1% FDR threshold in at least three replicates per group were retained, and the peak areas of the target fragment ions of those peptides were extracted across the experiments using an extracted-ion chromatogram (XIC) window of 4 minutes. Protein levels were estimated by summing all the transitions from all the peptides for a given protein [16] and normalized to the total intensity at the protein level.

The mass spectrometry proteomics data have been deposited to the ProteomeXchange Consortium via the PRIDE [17] partner repository with the dataset identifier PXD021455.

Statistical Analysis

To perform statistical comparisons between groups of samples to software SPSS v23 (IBM) was used. The non-parametric Mann Whitney U-test was used for all comparisons due to the reduced number of samples per comparison group or to the fact that the samples did not follow a normal distribution.

S1 Table 1 – Information about the windows used in SWATH acquisition.

Window	Start Mass (Da)	Stop Mass (Da)	Mass Interval (Da)	CES
Window 1	349.5	360.9	11.4	5
Window 2	359.9	375.2	15.3	5
Window 3	374.2	389.2	15	5
Window 4	388.2	402.2	14	5
Window 5	401.2	415.3	14.1	5
Window 6	414.3	427.4	13.1	5
Window 7	426.4	439.1	12.7	5
Window 8	438.1	449.9	11.8	5
Window 9	448.9	460.7	11.8	5
Window 10	459.7	471.1	11.4	5
Window 11	470.1	480.5	10.4	5
Window 12	479.5	490	10.5	5
Window 13	489	499	10	5
Window 14	498	508	10	5
Window 15	507	516.5	9.5	5
Window 16	515.5	525.1	9.6	5
Window 17	524.1	533.2	9.1	5
Window 18	532.2	540.8	8.6	5
Window 19	539.8	548.5	8.7	5
Window 20	547.5	555.7	8.2	5
Window 21	554.7	563.4	8.7	5
Window 22	562.4	570.6	8.2	5
Window 23	569.6	577.8	8.2	5

Window 24	576.8	585.4	8.6	5
Window 25	584.4	592.6	8.2	5
Window 26	591.6	600.3	8.7	5
Window 27	599.3	607.9	8.6	5
Window 28	606.9	615.6	8.7	5
Window 29	614.6	623.2	8.6	5
Window 30	622.2	630.9	8.7	5
Window 31	629.9	638.5	8.6	5
Window 32	637.5	646.2	8.7	5
Window 33	645.2	653.8	8.6	5
Window 34	652.8	661.5	8.7	5
Window 35	660.5	669.1	8.6	5
Window 36	668.1	677.2	9.1	5
Window 37	676.2	685.3	9.1	5
Window 38	684.3	693.9	9.6	5
Window 39	692.9	702.9	10	5
Window 40	701.9	711.9	10	5
Window 41	710.9	721.3	10.4	5
Window 42	720.3	731.2	10.9	5
Window 43	730.2	741.6	11.4	5
Window 44	740.6	752.4	11.8	5
Window 45	751.4	763.6	12.2	5
Window 46	762.6	775.8	13.2	5
Window 47	774.8	787.9	13.1	5
Window 48	786.9	800.5	13.6	8
Window 49	799.5	814.5	15	8
Window 50	813.5	829.3	15.8	8
Window 51	828.3	845.5	17.2	8
Window 52	844.5	865.3	20.8	8
Window 53	864.3	886.5	22.2	8
Window 54	885.5	911.2	25.7	8
Window 55	910.2	939.1	28.9	8

Window 56	938.1	972	33.9	8
Window 57	971	1008.4	37.4	10
Window 58	1007.4	1053.4	46	10
Window 59	1052.4	1120	67.6	10
Window 60	1119	1249.6	130.6	10

References

1. Doerr A. DIA mass spectrometry. Nature Methods. 2014;12:35. doi: 10.1038/nmeth.3234.
2. Domon B, Aebersold R. Options and considerations when selecting a quantitative proteomics strategy. Nature biotechnology. 2010;28(7):710-21. doi: 10.1038/nbt.1661. PubMed PMID: 20622845.
3. Bantscheff M, Schirle M, Sweetman G, Rick J, Kuster B. Quantitative mass spectrometry in proteomics: a critical review. Analytical and bioanalytical chemistry. 2007;389(4):1017-31. doi: 10.1007/s00216-007-1486-6. PubMed PMID: 17668192.
4. Gillet LC, Navarro P, Tate S, Röst H, Selevsek N, Reiter L, et al. Targeted data extraction of the MS/MS spectra generated by data-independent acquisition: a new concept for consistent and accurate proteome analysis. Molecular & cellular proteomics : MCP. 2012;11(6):O111.016717-O111. doi: 10.1074/mcp.O111.016717.
5. Craft GE, Chen A, Nairn AC. Recent advances in quantitative neuroproteomics. Methods. 2013;61(3):186-218. doi: 10.1016/j.ymeth.2013.04.008. PubMed PMID: 23623823; PubMed Central PMCID: PMC3891841.
6. Roemmelt AT, Steuer AE, Poetzsch M, Kraemer T. Liquid chromatography, in combination with a quadrupole time-of-flight instrument (LC QTOF), with sequential window acquisition of all theoretical fragment-ion spectra (SWATH) acquisition: systematic studies on its use for screenings in clinical and forensic toxicology and comparison with information-dependent acquisition (IDA). Analytical chemistry. 2014;86(23):11742-9. doi: 10.1021/ac503144p. PubMed PMID: 25329363.
7. Guo T, Kouvonen P, Koh CC, Gillet LC, Wolski WE, Rost HL, et al. Rapid mass spectrometric conversion of tissue biopsy samples into permanent quantitative digital proteome maps. Nat Med. 2015;21(4):407-13. doi: 10.1038/nm.3807. PubMed PMID: 25730263; PubMed Central PMCID: PMCPMC4390165.
8. Chen MX, Zhang Y, Fernie AR, Liu YG, Zhu FY. SWATH-MS-Based Proteomics: Strategies and Applications in Plants. Trends in biotechnology. 2020. Epub 2020/10/11. doi: 10.1016/j.tibtech.2020.09.002. PubMed PMID: 33036785.
9. Zhu FY, Song YC, Zhang KL, Chen X, Chen MX. Quantifying Plant Dynamic Proteomes by SWATH-based Mass Spectrometry. Trends in plant science. 2020;25(11):1171-2. Epub 2020/09/07. doi: 10.1016/j.tplants.2020.07.014. PubMed PMID: 32891562.
10. Anjo SI, Santa C, Manadas B. Short GeLC-SWATH: a fast and reliable quantitative approach for proteomic screenings. Proteomics. 2015;15(4):757-62. doi: 10.1002/pmic.201400221. PubMed PMID: 25418953.
11. Manadas B, Santos AR, Szabadfi K, Gomes JR, Garbis SD, Fountoulakis M, et al. BDNF-induced changes in the expression of the translation machinery in hippocampal

neurons: protein levels and dendritic mRNA. Journal of proteome research. 2009;8(10):4536-52. doi: 10.1021/pr900366x. PubMed PMID: 19702335.
12. Gillet LC, Navarro P, Tate S, Rost H, Selevsek N, Reiter L, et al. Targeted data extraction of the MS/MS spectra generated by data-independent acquisition: a new concept for consistent and accurate proteome analysis. Molecular & cellular proteomics : MCP. 2012;11(6):O111 016717. doi: 10.1074/mcp.O111.016717. PubMed PMID: 22261725; PubMed Central PMCID: PMC3433915.
13. Sennels L, Bukowski-Wills JC, Rappsilber J. Improved results in proteomics by use of local and peptide-class specific false discovery rates. BMC bioinformatics. 2009;10:179. doi: 10.1186/1471-2105-10-179. PubMed PMID: 19523214; PubMed Central PMCID: PMC2709624.
14. Tang WH, Shilov IV, Seymour SL. Nonlinear fitting method for determining local false discovery rates from decoy database searches. Journal of proteome research. 2008;7(9):3661-7. doi: 10.1021/pr070492f. PubMed PMID: 18700793.
15. Lambert JP, Ivosev G, Couzens AL, Larsen B, Taipale M, Lin ZY, et al. Mapping differential interactomes by affinity purification coupled with data-independent mass spectrometry acquisition. Nature methods. 2013;10(12):1239-45. doi: 10.1038/nmeth.2702. PubMed PMID: 24162924; PubMed Central PMCID: PMC3882083.
16. Collins BC, Gillet LC, Rosenberger G, Rost HL, Vichalkovski A, Gstaiger M, et al. Quantifying protein interaction dynamics by SWATH mass spectrometry: application to the 14-3-3 system. Nature methods. 2013;10(12):1246-53. doi: 10.1038/nmeth.2703. PubMed PMID: 24162925.
17. Vizcaino JA, Csordas A, Del-Toro N, Dianes JA, Griss J, Lavidas I, et al. 2016 update of the PRIDE database and its related tools. Nucleic Acids Res. 2016;44(22):11033. doi: 10.1093/nar/gkw880. PubMed PMID: 27683222; PubMed Central PMCID: PMCPMC5159556.
