# Supplementary material for: Disclosing proteins in the leaves of cork oak plants associated with the immune response to Phytophthora cinnamomi inoculation in the roots: A long-term proteomics approach
Source: PLoS One. 2021 Jan 22;16(1):e0245148. doi: 10.1371/journal.pone.0245148 (PMC7822296; doi:10.1371/journal.pone.0245148)
Supplement: Attachment — Submitted filename: PONE-D-20-29356_reviewer comments.docx [file pone.0245148.s011.docx]

PLOS ONE

#### Disclosing proteins in cork oak plants associated with the immune response to Phytophthora cinnamomi inoculation in a long-term assay

--Manuscript Draft--

| Manuscript Number: | PONE-D-20-29356 |
| --- | --- |
| Article Type: | Research Article |
| Full Title: | Disclosing proteins in cork oak plants associated with the immune response to Phytophthora cinnamomi inoculation in a long-term assay |
| Short Title: | Cork oak immune response to Phytophthora cinnamomi |
| Corresponding Author: | Ana Cristina Coelho, Ph.D. University of Algarve  Faro, PORTUGAL |
| Keywords: | Quercus suber; Phytophthora cinnamomi; quantitative proteomics; plant immune response; cork oak decline |
| Abstract: | The pathological interaction between oak trees and Phytophthora cinnamomi has implications in the cork oak decline observed over the last decades in the Iberian Peninsula. During host colonization, the phytopathogen secretes effector molecules like elicitins to increase disease effectiveness. The objective of this study was to unravel the proteome changes associated with the cork oak immune response triggered by P. cinnamomi inoculation in a long-term assay, through SWATH-MS quantitative proteomics performed in the oak leaves. Using the Arabidopis proteome database as a reference, 424 proteins have been confidently quantified in cork oak leaves, of which 80 proteins showed a p-value below 0.05 or a fold-change greater than 2 or less than 0.5 in their levels between inoculated and control samples being considered as altered. The inoculation of cork oak roots with P. cinnamomi increased the levels of proteins associated with protein-DNA complex assembly, lipid oxidation, response to endoplasmic reticulum stress, and pyridine-containing compound metabolic process in the leaves. In opposition, several proteins associated with cellular metabolic compound salvage and monosaccharide catabolic process had significantly decreased abundances. The most significant abundance variations were observed for the Ribulose 1,5-Bisphosphate Carboxylase small subunit (RBCS1A), Heat Shock protein 90-1 (Hsp90-1), Lipoxygenase 2 (LOX2) and Histone superfamily protein H3.3 (A8MRLO/At4G40030) revealing a pertinent role for these proteins in the host- pathogen interaction mechanism. This work represents the first SWATH-MS analysis performed in cork oak plants inoculated with P. cinnamomi and highlights host proteins that have a relevant action in the homeostatic states that emerge from the interaction between the oomycete and the host in the long term and in a distal organ. |
| Order of Authors: | Ana Cristina Coelho, Ph.D. |
|  | Rosa Pires |
|  | Gabriela Schütz |
|  | Cátia Santa |
|  | Bruno Manadas |
|  | Patrícia Pinto |
| Additional Information: |  |
| Question | Response |
| Financial Disclosure  Enter a financial disclosure statement that describes the sources of funding for the work included in this submission. Review the [submission guidelines](http://journals.plos.org/plosone/s/submission-guidelines#loc-financial-disclosure-statement) for detailed | This work was financially supported by FCT, integrated in projects UID/Multi/00631/2013, UID/Multi/00631/2019 and UIDB/00631/2020 CEOT BASE to CEOT and ACC, GS and RP; UIDB/04326/2020 to CCMAR; fellowship SFRH/BPD/84033/2012 and researcher contract with the University of Algarve under Norma Transitória-DL57/2016/CP1361/CT0015 to PP; contract NIBAP (ALG-01-0247- FEDER-037303) to RP; projects POCI-01-0145-FEDER-007440 (Ref. |

*Powered by Editorial Manager® and ProduXion Manager® from Aries Systems Corporation*

| requirements. View published research articles from [PLOS ONE](http://journals.plos.org/plosone/) for specific examples.  This statement is required for submission and will appear in the published article if the submission is accepted. Please make sure it is accurate. | UIDB/04539/2020), POCI-01-0145-FEDER-016428 (Ref. SAICTPAC/0010/2015), POCI-01-0145-FEDER-029311 (Ref. PTDC/BTM-TEC/29311/2017), POCI-01-0145- FEDER-30943 (Ref. PTDC/MECPSQ/30943/2017) and PTDC/MED-NEU/27946/2017  to CNC, BM and CS. The work at CNC was also funded by the National Mass Spectrometry Network (RNEM) under contract POCI-01-0145-FEDER-402-022125 (Ref. ROTEIRO/0028/2013).  The funders had no role in study design, data collection and analysis, decision to publish, or preparation of the manuscript. |
| --- | --- |
| Unfunded studies  Enter: The author(s) received no specific funding for this work.  Funded studies  Enter a statement with the following details:   - Initials of the authors who received each award - Grant numbers awarded to each author - The full name of each funder - URL of each funder website - Did the sponsors or funders play any role in the study design, data collection and analysis, decision to publish, or preparation of the manuscript? - NO - Include this sentence at the end of your statement: The funders had no role in study design, data collection and analysis, decision to publish, or preparation of the manuscript. - YES - Specify the role(s) played. |  |
| * typeset |  |
| Competing Interests  Use the instructions below to enter a competing interest statement for this submission. On behalf of all authors, disclose any [competing interests](http://journals.plos.org/plosone/s/competing-interests) that could be perceived to bias this work—acknowledging all financial support and any other relevant financial or non- financial competing interests.  This statement will appear in the published article if the submission is accepted. Please make sure it is accurate. View published research articles from [PLOS ONE](http://journals.plos.org/plosone/) for specific examples. | The authors have declared that no competing interests exist. |

*Powered by Editorial Manager® and ProduXion Manager® from Aries Systems Corporation*

| NO authors have competing interests  Enter: The authors have declared that no competing interests exist.  Authors with competing interests  Enter competing interest details beginning with this statement:  I have read the journal's policy and the authors of this manuscript have the following competing interests: [insert competing interests here] |  |
| --- | --- |
| * typeset |  |
| Ethics Statement  Enter an ethics statement for this submission. This statement is required if the study involved:   - Human participants - Human specimens or tissue - Vertebrate animals or cephalopods - Vertebrate embryos or tissues - Field research   Write "N/A" if the submission does not require an ethics statement.  General guidance is provided below. Consult the [submission guidelines](http://journals.plos.org/plosone/s/submission-guidelines#loc-guidelines-for-specific-study-types) for detailed instructions. Make sure that all information entered here is included in the Methods section of the manuscript. | N/A |

*Powered by Editorial Manager® and ProduXion Manager® from Aries Systems Corporation*

| Format for specific study types  Human Subject Research (involving human participants and/or tissue)   - Give the name of the institutional review board or ethics committee that approved the study - Include the approval number and/or a statement indicating approval of this research - Indicate the form of consent obtained (written/oral) or the reason that consent was not obtained (e.g. the data were analyzed anonymously)   Animal Research (involving vertebrate animals, embryos or tissues)   - Provide the name of the Institutional Animal Care and Use Committee (IACUC) or other relevant ethics board that reviewed the study protocol, and indicate whether they approved this research or granted a formal waiver of ethical approval - Include an approval number if one was obtained - If the study involved non-human primates, add additional details about animal welfare and steps taken to ameliorate suffering - If anesthesia, euthanasia, or any kind of animal sacrifice is part of the study, include briefly which substances and/or methods were applied   Field Research  Include the following details if this study involves the collection of plant, animal, or other materials from a natural setting:   - Field permit number - Name of the institution or relevant body that granted permission |  |
| --- | --- |
|  |  |
| Data Availability  Authors are required to make all data underlying the findings described fully available, without restriction, and from the time of publication. PLOS allows rare exceptions to address legal and ethical concerns. See the [PLOS Data Policy](http://journals.plos.org/plosone/s/data-availability) and [FAQ](http://journals.plos.org/plosone/s/data-availability#loc-faqs-for-data-policy) for detailed information. | Yes - all data are fully available without restriction |

*Powered by Editorial Manager® and ProduXion Manager® from Aries Systems Corporation*

| A Data Availability Statement describing where the data can be found is required at submission. Your answers to this question constitute the Data Availability Statement and will be published in the article, if accepted. |  |
| --- | --- |
| Important: Stating ‘data available on request from the author’ is not sufficient. If your data are only available upon request, select ‘No’ for the first question and explain your exceptional situation in the text box. |  |
| Do the authors confirm that all data underlying the findings described in their manuscript are fully available without restriction? |  |
| Describe where the data may be found in full sentences. If you are copying our sample text, replace any instances of XXX with the appropriate details.   - If the data are held or will be held in a public repository, include URLs, accession numbers or DOIs. If this information will only be available after acceptance, indicate this by ticking the box below. For example: All XXX files are available from the XXX database (accession number(s) XXX, XXX.). - If the data are all contained within the manuscript and/or Supporting Information files, enter the following: All relevant data are within the manuscript and its Supporting Information files. - If neither of these applies but you are able to provide details of access elsewhere, with or without limitations, please do so. For example:   Data cannot be shared publicly because of [XXX]. Data are available from the XXX Institutional Data Access / Ethics Committee (contact via XXX) for researchers who meet the criteria for access to confidential data.  The data underlying the results presented in the study are available from (include the name of the third party | The mass spectrometry proteomics data have been deposited to the ProteomeXchange Consortium via the PRIDE partner repository with the dataset identifier PXD021455.  Submission details:  Project Name: Disclosing proteins in cork oak plants associated with the immune response to P. cinnamomi inoculation in a long-term assay  Project accession: PXD021455 Project DOI: Not applicable Reviewer account details:  Username: [reviewer_pxd021455@ebi.ac.uk](mailto:reviewer_pxd021455@ebi.ac.uk) Password: oykuhFsN |

*Powered by Editorial Manager® and ProduXion Manager® from Aries Systems Corporation*

| and contact information or URL).   - This text is appropriate if the data are owned by a third party and authors do not have permission to share the data.   * typeset |  |
| --- | --- |
| Additional data availability information: | Tick here if the URLs/accession numbers/DOIs will be available only after acceptance of the manuscript for publication so that we can ensure their inclusion before publication. |

*Powered by Editorial Manager® and ProduXion Manager® from Aries Systems Corporation*

Manuscript	[Click here to access/download;Manuscript;Manuscript_Coelho et al_2020_PLoS ONE.docx](https://www.editorialmanager.com/pone/download.aspx?id=27432586&amp;guid=09f5d815-d018-42f6-87b0-bd8fd47cfef1&amp;scheme=1)

1

Disclosing proteins in cork oak plants associated with the immune response to

*Phytophthora cinnamomi* inoculation in a long-term assay

2

3 Ana Cristina Coelho(1,2)*¶, Rosa Pires(1)#, Gabriela Schütz(1,3)&, Cátia Santa(4,6)⁕, Bruno
4 Manadas(4)⁕, Patrícia Pinto(5)&

5

6

7 1 Center for Electronic, Optoelectronic and Telecommunications (CEOT), University of
8 Algarve, Faro, Portugal

9

10 2 Escola Superior de Educação e Comunicação (ESEC), University of Algarve, Faro,
11 Portugal

12

13	3 Instituto Superior de Engenharia, University of Algarve, Faro, Portugal

14

15 4 CNC - Center for Neuroscience and Cell Biology, University of Coimbra, Coimbra,
16 Portugal;

17

18	5 Center for Marine Sciences (CCMAR), University of Algarve, Faro, Portugal

19

20 6 Institute for Interdisciplinary Research (IIIUC), University of Coimbra, Coimbra,
21 Portugal.

22

23 *Corresponding author:
24 E-[mail: acoelho@ualg.pt](mailto:acoelho@ualg.pt) (ACC)

25

26 ¶ACC: conceptualization, funding acquisition, investigation, methodology, data curation,
27 project administration, resources, supervision, visualization and writing-original draft.
28 &GS and PP: data curation, formal analysis, writing-review and editing.
29 ⁕BM and CS: data curation, formal analysis, writing-review, editing and funding
30 acquisition.
31 #RP: investigation, writing-review and editing.

32

# 33 Abstract

34 The pathological interaction between oak trees and *Phytophthora cinnamomi* has
35 implications in the cork oak decline observed over the last decades in the Iberian
36 Peninsula. During host colonization, the phytopathogen secretes effector molecules like
37 elicitins to increase disease effectiveness. The objective of this study was to unravel the
38 proteome changes associated with the cork oak immune response triggered by *P.*
39 *cinnamomi* inoculation in a long-term assay, through SWATH-MS quantitative
40 proteomics performed in the oak leaves. Using the *Arabidopis* proteome database as a
41 reference, 424 proteins were confidently quantified in cork oak leaves, of which 80
42 proteins showed a p-value below 0.05 or a fold-change greater than 2 or less than 0.5 in
43 their levels between inoculated and control samples being considered as altered. The
44 inoculation of cork oak roots with *P. cinnamomi* increased the levels of proteins
45 associated	with	protein-DNA	complex	assembly, lipid oxidation, response to
46 endoplasmic reticulum stress, and pyridine-containing compound metabolic process in
47 the leaves. In opposition, several proteins associated with cellular metabolic compound
48 salvage and monosaccharide catabolic process had significantly decreased abundances.
49 The most significant abundance variations were observed for the Ribulose 1,5-
50 Bisphosphate Carboxylase small subunit (RBCS1A), Heat Shock protein 90-1 (Hsp90-
51 1),	Lipoxygenase	2	(LOX2)	and	Histone	superfamily	protein	H3.3
52 (A8MRLO/At4G40030) revealing a pertinent role for these proteins in the host-pathogen
53 interaction mechanism. This work represents the first SWATH-MS analysis performed in
54 cork oak plants inoculated with *P. cinnamomi* and highlights host proteins that have a
55 relevant action in the homeostatic states that emerge from the interaction between the
56 oomycete and the host in the long term and in a distal organ.

57

# 58 Introduction

59 The soil-borne oomycete *Phytophthora cinnamomi* infects the roots of cork oak
60 plants, induces necrotic lesions, and the loss of fine roots [1, 2]. This evidence,
61 combined with other factors, are the hallmark for the decline of the cork oak savanna-like
62 ecosystem (*Quercus suber*; cork oak *montado*) in Portugal. Climate change is reducing
63 water availability (drought) [3], and the effectiveness of roots in absorbing water is
64 affected by the health status of the plant [4, 5], which can become less effective in
65 accessing groundwater during drought [6]. Insect colonization [7] and fungal
66 infections [8, 9] can weaken the tree's defence system and thus contribute to the decline.
67 To help maintain the sustainability of the cork oak agro-forests, the recommended
68 focus is to adopt good management practices [10].
69 During inter and intracellular cork oak colonization by *P. cinnamomi*, small 10
70 kDa proteins (elicitins) are secreted by the oomycete and increases disease effectiveness.
71 This has been demonstrated by studying a β-cinnamomin silenced *P. cinnamomi* strain,
72 which acted as a weaker pathogen against cork oak when compared to the virulence
73 revealed by the wild type [11, 12]. In the roots of the narrow-leafed lupin (*Lupinus*
74 *angustifolius*) infected with *P. cinnamomi*, the expression of *β*-cinnamomin starts to be
75 detected as early as 24 h post-inoculation, and follows the development of the mycelium
76 into the host, anchored to a mycelial cell wall protein, emphasizing the recognition of
77 these proteins as virulence factors [13]. However, effector molecules from the RxLR,
78 CRN (for Crinkling and Necrosis) and	Nep1-like (NLPs) protein families are also
79 potentially secreted, encoded by the 171 RxLR, 72 NLPs and 29 CRN putative genes
80 present in the genomes (78 Mb) of three *P. cinnamomi* isolates, being able to suppress or
81 bypass the plant basic defence responses [14]. In the compatible interaction between the
82 red-flowered moort (*Eucalyptus nitans*) and *P. cinnamomi* a putative CRN1 effector was
83 the highest expressed gene *in planta* [15]. The molecular mechanisms by which the
84 effector molecules act are largely unknown, although the entry of some effector proteins
85 into the plant host cells is known to follow a mechanism of endocytosis after binding to
86 receptor molecules of phosphatidylinositol-3-phosphate (PI-3-P) mediated by the RxLR
87 domain [16, 17]. In the nucleus, the effectors control reactions that trigger host cell death
88 or hypersensitive responses (HR) [18, 19], and in the nucleolus, they can act as
89 modulators of histone acetyltransferases (HAT) to reprogram the plant defence gene
90 expression and promote infection [20].
91 Following compatible or incompatible reactions with plants, oomycete
92 compounds like lipids or carbohydrates referred to as Pathogen-Associated Molecular
93 Patterns (PAMPs) and effector biomolecules elicit local resistance responses or
94 PAMPs/effector-triggered immunity (PTI/ETI)) in their hosts [21]. In *Q. suber* root cells,
95 during the first 24 h of interaction with *P. cinnamomi*, metabolic patterns undergo a non-
96 linear variation for compounds with carbohydrate, glycoconjugate and lipid groups
97 detected at 2 h, 8 h, and 24 h post-inoculation; however, these were indistinguishable
98 from mock-inoculated plants at 16 h, the point at which the pattern reverses [22]. This
99 reversal point may indicate the moment from which the host's defence response is effective
100 after recognition of the pathogen. At the transcriptomic level, the expression of genes
101 encoding pathogenesis-related proteins like *PR-1*, chitinase (*PR-3*), chitin-binding
102 protein (*PR-4*), thaumatin-likeprotein/osmotin (*PR-5*) and peroxidases *(PR-9)* is up-
103 regulated in the stem tissues of *P. cinnamomi*-infected *Eucalyptus nitens* compared with
104 control plants, 5 days post-inoculation [15]. Differential gene expression for defence
105 associated proteins PR-5, LOX (lipoxygenase), endochitinase, glutathione-S-transferase
106 and metallothionein was also observed in avocado roots challenged with *P. cinnamomi*
107 [23]. In a more detailed analysis of the transcriptome of chestnut roots inoculated with *P.*
108 *cinnamomi*, the multiplicity of the defence responses becomes evident with the
109 identification of genes related to the HR (hypersensitive response), cell wall
110 strengthening, synthesis of flavonoids and systemic acquired resistance [24]. Further,
111 resistance (R) genes coding to transmembrane proteins such as LRR receptor-like
112 serine/threonine-protein kinase in two *Castanea* species [24] and CC-NB-LRR (coiled
113 coil-nucleotide binding-leucine rich repeat) in cork oak [25], are also potentially
114 associated to the recognition of effector molecules, eventually interacting, according to
115 the gene-for-gene model [26]. Activation of these resistance proteins can result in the
116 activation of mitogen-activated protein kinase (MAPK) signal transduction cascades,
117 leading to transcription factor activation and transcription of responsive genes, and these
118 cascades can also be activated by proteins sensitive to the production of reactive oxygen
119 species (ROS, O2-, H2O2) [reviewed by 21, 27-29].
120 Salicylic acid (SA)/salicylate is also a signaling molecule that plays a central role
121 in PAMPs/effector-triggered immunity (PTI/ETI) and in the systemic acquired resistance
122 (SAR), it is a type of immunity that extends to the entire plant beyond the site of infection,
123 protecting the plant against a broad spectrum of pathogens [30, 31]. The expression of a
124 large number of pathogenesis-related genes is activated by nuclear transcription factors
125 interacting with NPR1 monomers (nonexpressor of pathogenesis related 1), known as the
126 main regulatory molecule of the SA-signaling pathway [32, 33]. *P. sojae* uses as a
127 pathogenic strategy the secretion of isochorismatases as effectors that interfere with the
128 accumulation of SA, suppressing precursors in the salicylate metabolism pathway and,
129 thus, inhibiting plant defence responses [34]. Antagonistically, the protein encoded by the
130 gene *OPEL* of *P. parasitica* enhances the expression of SA-responsive genes, like *PAL*,
131 *PR1* and *PR5* and induces SAR in *Nicotiana tabacum* with evidence for long-distance
132 signaling, acting more as an elicitor of defence responses in this plant species [35].
133 Due to the complexity of the innate response, some authors have attempted to
134 show in a comprehensive and schematic way the defence mechanisms activated during
135 the interaction of *P. cinnamomi* or *Phytophthora* spp. with woody plant species by creating
136 hypothetical molecular models in which the different pathways are annotated [28, 36, 37].
137 To overcome the harmful implications of *P. cinnamomi* on susceptible species of thousands of
138 plants worldwide, one of the current challenges is the identification of molecular
139 markers or physiological processes suitable for recognition of resistant or susceptible host
140 plant species or varieties. Information about the constitutive expression level of
141 pathogenesis-related genes in non-infected hosts and the reaction time mediating the
142 recognition of the invader and the activation of local and systemic defence systems can
143 contribute to this global goal, and was decisive for the recognition of *Castanea crenata*
144 as a less susceptible species than *C. sativa* [28]. In less susceptible avocado rootstocks,
145 the physical and chemical composition of the host's tissues at the site of infection was
146 critical to the effectiveness of *P. cinnamomi* zoospore germination and penetration, as the early
147 deposition of callose instead of lignin near the site of hyphae penetration along the cell
148 wall hindered the development of the oomycete’s hyphae [38].
149 The hypothesis of the present study is that after inoculation of plant roots with a pathogen,
150 an immune response is initiated that will lead to a new homeostatic state, with protein
151 changes that can be detectable in the long-term, distally from the infection site. The aim
152 was to identify and quantify proteins by SWATH-MS quantitative
153 proteomics [39] in the leaves from cork oak plants inoculated in the roots with *P. cinnamomi* and
154 from non-inoculated plants, at 248 days post-inoculation.
155 The leaves are a distal organ that can be sampled in a non-invasive way, so they can also
156 be a potential organ for practical monitoring of infection or resistance. Four hundred and
157 twenty-four proteins were identified in the cork oak leaves, and a subset of 80 proteins
158 showed differential levels between inoculated and control plants, being considered
159 responsive to *P. cinnamomi*. These included 18 proteins associated with several gene
160 ontologies (GO) biological processes, and their potential role in the cork oak immune
161 response is discussed. The GO cellular component “stromules” was also significantly
162 enriched among the differential proteins, indicating that communication between cellular
163 organelles may be important in the cork oak immune response to *P. cinnamomi*.
164 To our knowledge, this is the first report on the proteome changes associated to
165 the cork oak immune response triggered by *P. cinnamomi*.

# 167 Materials and Methods

168 The design of the project included several experimental procedures operated at
169 different time points, and Fig 1 shows the different phases, schematically. In the first
170 phase, the biological material was prepared consisting of twelve cork oak seedlings,
171 germinated from seeds, with half of these plants being inoculated with *P. cinnamomi*. The
172 following phases started 248 days after inoculation and included the harvesting of the
173 leaves from each plant for protein extraction and subsequent SWATH-MS proteomics.
174 The experimental assay ended with the bioinformatic annotation and quantification of
175 proteins present in the extracts of each plant.
177 **Fig 1. Experimental design**. Reference to the main procedural phases of the project.

## 179 Biological material

180 Cork oak plants used in this experimental project were germinated from acorns taken from
181 six cork oak trees located in Cachopo, Algarve, Portugal. Parental cork
182 oak trees referenced as S1.1, S2.1, S4.1, S5.1, S7.1, and S8.1 showed signs of decline at
183 distinct development stages, based on visual observation of the canopy defoliation level typical of *P. cinnamomi* infection.
184 The study included two experimental conditions: 6 cork oak plants inoculated with the
185 PA45 *P. cinnamomi* isolate and 6 non-inoculated plants. Seeds from six parental cork oak
186 trees were germinated and were distributed between the control and inoculated groups so that
187 each inoculated plant had a paired control from the same progenitor (Table 1).

##### 189 Table 1. References for cork oak plants.

| Cork oak treesa  Acorn progenitors | Control plants (C)a | *P. cinnamomi* inoculated plants   (I) a |
| --- | --- | --- |
| S1.1b | S1.1.eb | S1.1.1b |
| S2.1 | S2.1.3 | S2.1.1 |
| S4.1 | S4.1.3 | S4.1.2 |
| S5.1 | S5.1.4 | S5.1.1 |
| S7.1 | S7.1.4 | S7.1.3 |
| S8.1 | S8.1.3 | S8.1.1 |

190 aThe plants used in the experimental assay come from 6 parental cork oak trees, appearing
191 in pairs, one in the control group (C) and the other in the inoculated group (I).
192 bExample: Cork oak tree S1.1 produced the acorns that gave rise to the plants S1.1.e (C)

193	and S1.1.1 (I).

194

195 The inoculation procedures described by Horta et al. 2010 [11] were followed.
196 Briefly, a xx mm^2^ diameter *P. cinnamomi* mycelium isolate PA45, grown in solid agar, directly contacted
197 with root tissues of seventy-seven day-hold cork oak plantlets. Forty-eight hours after *P.*
198 *cinnamomi* inoculation all plants were potted into xxx substrate in free-draining plastic containers (dimensions), transferred outside and
199 watered regularly to container capacity. ~~The experimental assay lasted for 248 days, more than 8 months.~~
200 S1 Fig outlines the procedure and timing of the experiment.
201 After 248 days, or more than 8 months, the leaves of cork oak plants, inoculated and non-inoculated, were
202 collected and immediately frozen in liquid nitrogen, and
203 stored at -80ºC until further use for protein extraction.

| 204 |  |
| --- | --- |
| 205 | **SWATH-MS Proteomics** |
| 206 | **Total protein extraction** |
| 207 | The optimized extraction of proteins from cork oak leaves included eight steps. |

208 1) Leaf tissue (200 mg) was ground in a mortar and pestle in the
209 presence of liquid nitrogen to obtain a fine powder. 2) Buffer 1 (1.25 mL/100 mg sample;
210 DTT –Dithiothreitol 60 mM; 10% TCA-Trichloroacetic acid solubilized in acetone) was
211 added to the mortar and samples were macerated in the presence of the buffer with the
212 pestle. 3) The heterogeneous solution was transferred to a 2 mL microcentrifuge tube and
213 incubated for 1 hour at -80 ºC. 4) The samples were centrifuged at 15,000× g for 15 min
214 at 4 ºC and the supernatant was discarded. 5) The pellets were dissolved in 1 mL of Buffer
215 2 (2.5 ml/100 mg sample; DTT 60 mM solubilized in acetone), and the sample volume
216 was divided into two microcentrifuge tubes followed by the addition of 750 µL of Buffer
217 2 to each tube. 6) These solutions were incubated for 1 hour at -80 ºC. Procedures 5 and
218 6 were repeated until the solution was clear green. 7) The samples were centrifuged at
219 15,000×g for 15 min at 4 ºC and the supernatant was discarded. 8) Pellets were dried and
220 resuspended in 250 µL of SDS-PAGE buffer [TRIS Glycine buffer solution (25 mM
221 TRIS; 192 mM Glycine; Sigma-Aldrich); 2% SDS-Sodium dodecyl sulfate] followed by
222 incubation at 95 ºC for 5 min and centrifugation at 20,000x g for 15 min at 4 ºC. All
223 reagents used were molecular biology grade.
224 Protein concentration in the samples was estimated using the 2D-Quant kit (GE
225 Healthcare Life Sciences) with serum albumin as standard [40].
227 **SWATH-MS strategy**
228 For the proteomic screening, the short GeLC-SWATH-MS strategy was used
229 according to [41] with minor modifications. Briefly, 50 μg of each sample and a pooled
230 sample per group (pool of the protein extracts for the six control or six inoculated
231 samples) were subjected to *in-gel* digestion after a partial SDS-PAGE run. Then, LC-MS
232 information was acquired in two different acquisition modes: information-dependent
233 acquisition (IDA) of the pooled samples, and SWATH-MS (Sequential Windowed data-
234 independent Acquisition of the Total High-resolution Mass Spectra) ~~acquisition~~ of each
235 sample.	Protein	identification	and	library	construction	was	performed	using
236 ProteinPilot™ (v5.0.1, Sciex), and compared with the *Arabidopsis thaliana* reference
237 proteome (retrieved from htt[ps://www.uniprot.org](http://www.uniprot.org/)/ in April 2018). The relative
238 quantification was performed using the SWATH™ processing plug-in for PeakView™
239 (v2.2, Sciex). For each experimental group, the average protein levels, standard deviation
240 and percentage coefficient of variation (% CV) were calculated based on the quantification levels
241 obtained for each individual with eight replicates per group. The fold change (FC)
242 between inoculated and control plants was calculated by dividing the respective median
243 protein levels for all quantified proteins. Statistical comparisons between protein levels
244 were carried out using the software SPSS v23 (IBM) and the non-parametric Mann
245 Whitney U-test (MW). Proteins were considered as differentially modified when FC was
246 greater than 2 or less than 0.5 or MW p-value was below 0.05.
247 The	mass	spectrometry	proteomics	data	have	been	deposited	to	the
248 ProteomeXchange Consortium via the PRIDE [42] partner repository with the dataset
249 identifier PXD021455. A detailed description of LC-MS materials and methods are
250 provided as supporting information (S1 File).

### 252 Enrichment analyses and hierarchical clustering

253 Gene ontology (GO) and pathway (KEGG and Reactome) enrichment analyses
254 were carried out as in [43, 44], using Cytoscape v3.5.1 and ClueGO plug-in v2.5.2 [45,
255 46], comparing the list of 80 differentially modified proteins against the *Arabidopsis*
256 *thaliana* [organism 3702] set of GO biological process and cellular component databases
257 from November 2017. The following settings were used for the ClueGO enrichment
258 analysis (right-side): GO levels 3 to 8, Benjamini-Hochberg false discovery rate (FDR)
259 correction with a cut-off at FDR<0.05 and minimum of three genes/4% for terms to be
260 considered significant. The initial group size was set as 1, group merging at 50%, and
261 Kappa-statistics score at 0.4.

| 262 | Enrichment scores of the functionally related network groups were calculated as - |
| --- | --- |
| 263 | Log2 [group FDR]. The leading terms of each enriched group were those with the lowest |
| 264 | term FDR (highest enrichment score), which was used to name the respective group. |

265 The hierarchical clustering of the 80 differentially modified proteins was analysed
266 with Cluster 3.0 at [http://bonsai.hgc.jp/~mdehoon/software/cluster/software.htm#ctv](http://bonsai.hgc.jp/%7Emdehoon/software/cluster/software.htm#ctv)
267 [47] using normalized protein levels, applying the uncentered correlation and complete
268 linkage options.

# 270 Results and Discussion

## 271 Observation of the plants

272 Cork oak plants were in contact with the *P. cinnamomi* mycelium at the beginning
273 of the experiment for 48 hours, with no (re)inoculation over the next eight
274 months until the end of the experimental assay. During the first 24 hours of inoculation
275 with *P. cinnamomi* strain PA45, the aerial apex of the plant wilted and after 48 hours,
276 all the inoculated roots appeared necrotic at the inoculation site. Fig 2 illustrates these
277 visual changes observed for a representative cork oak during the inoculation time (a, b)
278 and seven months after inoculation, 1 month before the end of the experiment (c). At
279 this time, it was not possible to distinguish control plants from plants inoculated with
280 *P. cinnamomi* by visual observation of the aerial part. The vegetative development of the
281 plants was similar in both experimental conditions, inoculated and non-inoculated.

##### 283 Fig 2. Visual observation of the plants over the duration of the experiment. Images

284 taken at 24 hours during *P. cinnamomi* inoculation (a) and at 48 hours, ending inoculation
285 time, (b) and seven months post-inoculation (c)**.** (a) During the inoculation time, the
286 mycelium of *P. cinnamomi* PA45 isolate, grown on solid agar, was in direct contact with
287 root tissues of the cork oak for 48 hours (shown for the representative plant S8.1.1). (b)
288 The aerial apex of the plants wilted and the inoculated tissue appeared necrotic at the
289 inoculation site (white arrows). (c)	Seven months after inoculation, the
290 inoculated cork oak plants could not be differentiated from the non-inoculated based on
291 the observation of the foliage.
293 The virulence of the PA45 strain had been previously tested in cork oak roots,
294 inoculated under the same conditions as in the present study for 3 days [11]. Histological
295 studies performed on colonized root tissue demonstrated the ability of the oomycete to
296 invade the epidermis, cortical parenchyma and vascular cylinder both inter–and intra-
297 cellularly, and to destroy host cells [11].
298 In nature, at infested sites, cork oak trees may succumb (sudden death) after the
299 summer, without showing obvious previous symptoms of decline, or they can remain for
300 years with symptoms of defoliation that slowly worsen over time (slow decline). *P.*
301 *cinnamomi* has been isolated from roots of declined symptomatic trees and from infested
302 soils throughout Portugal [48], and it is important to recognize that oomycete infection
303 can be a determining factor for cork oak decline. However, the recovery of *P. cinnamomi*
304 from declining trees does not provide information about the plants' responsiveness or vitality over time.
305 At the end of this experiment, the plants inoculated with *P. cinnamomi* in the form of a
306 single event were visually asymptomatic for leaf fall or yellowing, plant height or number
307 of leaves. Nevertheless, the molecular interaction between *P. cinnamomi* and the hosts
308 may have occurred differently in each of the six plants due to the high molecular diversity
309 characteristic of *Q. suber* species [49]. One of the pertinent questions is how to detect that
310 a plant is or has been invaded/infected when it has no symptoms, avoiding the
311 (re)isolation of the pathogen and the use of invasive methods. As the degree of tree
312 defoliation is a symptom of decline and leaf harvesting is not invasive to the plants, the
313 search for molecular markers in the leaves of plants challenged with *P. cinnamomi* can
314 be a valuable option. The defence responses induced in the host by *P. cinnamomi* in the
315 long term and distant from the inoculation site establish a homeostatic state adapted to
316 living with the invader. This new homeostatic state stands out when comparing (below)
317 the type and amount of proteins present in the leaves of inoculated and non-inoculated
318 plants.
320 **Leaf cork oak proteome changes in response to *P. cinnamomi***

## 321 inoculation

322 SWATH-MS Proteomics analysis performed on leaf samples was used to
323 characterize the proteome of cork oak plants inoculated with *P. cinnamomi,* and compared
324 with the proteome of non-inoculated plants. With this technique, 12 individual protein
325 profiles were obtained, and protein abundances were quantified in each of the leaf
326 extracts. Thus, each replicate protein profile can reflect the genetic variability known for
327 *Q. suber* species, assuming the average of the results as a value closer to reality, but
328 providing the value of six replicate proteomic profiles for each of the two conditions
329 (control and inoculated). Three reference proteome databases were initially used for a
330 comprehensive protein sequence catalogue and to compare their differential abundance.
331 Table 2 shows the number of identified proteins and the number of quantified proteins in
332 the samples with reference to the Plant (containing all plant entries in the Uniprot
333 database), *Populus trichocarpa* and *Arabidopsis thaliana* protein databases (S1 File). To
334 continue with the data analysis, only the *Arabidopsis* (Uniprot) protein database was
335 chosen as a reference due to the robustness of information associated with proteins and
336 the lower frequency of proteins with unknown function.

##### 338 Table 2. Number of proteins identified and quantified.

| Number of identified proteins  (5% local-FDR)a | Reference proteome database | Number of quantified proteins  (5% local-FDR) |
| --- | --- | --- |
| 802 | Plant  (SwissProt database; April 2018) | 523 |
| 783 | *Populus trichocarpa*  (UP000006729; April 2018) | 536 |
| 609 | *Arabidopsis thaliana*  (UP000006548; April 2018) | 424 |

339 aA local false discovery rate of 5% was used as criteria for acceptance of peptide
340 assignments and protein identifications.
342 Using the *Arabidopis* proteome database as reference, 424 proteins were
343 confidently quantified in the cork oak leaves (S1 Table). From these, 80 proteins showed
344 a fold-change greater than 2 (or less than 0.5, in the case of proteins with decreased levels)
345 or a p-value below 0.05 in their median levels between inoculated and control samples
346 (Table 3). The Venn diagram in Fig 3 shows the number of proteins that met one or both
347 criteria.
349 **Fig 3. Protein groups.** The Venn diagram illustrates the number of proteins with a fold-
350 change greater than 2 or less than 0.5, those with a p-value below 0.05, and those that
351 meet both criteria based on protein levels between inoculated and control samples.
353 Among the 80 proteins with differential levels, 60 proteins increased abundance,
354 and 20 proteins decreased abundance in the leaves of experimental cork oak, 8 months
355 after *P. cinnamomi* inoculation, compared to the control plants (Table 3).

##### 357 Table 3. List of differentially accumulated proteins in *Q. suber* leaf proteome 8

358 **months after *P. cinnamomi* inoculation, using the *Arabidopis* proteome database as**

##### 359 a reference.

| ***Arabidopis***  **UniProt accessiona** | | **Median C (x10^-3^)b** | **Median I (x10^-3^)b** | **p≤0.05d** | **log2FC^c^** | **Protein namea** | **Protein Initialsa** |
| --- | --- | --- | --- | --- | --- | --- | --- |
|  | ***Proteins more abundant in P. cinnamomi inoculated samples compared to the control*** | | | | | | |
| P27323 | | 0.056 | 0.169 | 0.015 | 1.7 | Heat shock protein 90-1 | HS90-1 |
| Q9FIF3 | | 0.120 | 0.274 | 0.041 | 1.4 | 40S ribosomal protein S8-2 | RS82 |
| O81644 | | 0.020 | 0.037 | 0.132 | 1.3 | Villin-2 | VILI2 |
| P38418 | | 0.188 | 0.529 | 0.24 | 1.2 | Lipoxygenase 2, chloroplastic | LOX2 |
| A0A1P8AWT7 | | 0.533 | 1.361 | 0.041 | 1.1 | Catalase 3 | A0A1P8AWT7 |
| Q940B0 | | 0.232 | 0.436 | 0.065 | 1.1 | 60S ribosomal protein L18-3 | RL183 |
| A8MRL0 | | 0.183 | 0.397 | 0.015 | 1.1 | Histone superfamily protein | A8MRL0 At4G40030 |
| Q9FGX1 | | 0.124 | 0.260 | 0.004 | 0.9 | ATP-citrate synthase beta chain  protein 2 | ACLB2 |
| O04499 | | 0.098 | 0.195 | 0.009 | 0.9 | 2,3-bisphosphoglycerate- independent phosphoglycerate  mutase 1 | PMG1/iPGAM |
| O49485 | | 0.575 | 1.064 | 0.041 | 0.9 | D-3-phosphoglycerate  dehydrogenase 1, chloroplastic | SERA1 |
| Q9LF37 | | 0.038 | 0.074 | 0.041 | 0.9 | Chaperone protein ClpB3,  chloroplastic | CLPB3 |
| Q9STX5 | | 0.175 | 0.389 | 0.041 | 0.9 | Endoplasmin homolog | ENPL |
| Q9M040 | | 0.171 | 0.336 | 0.009 | 0.8 | Pyruvate decarboxylase 4 | PDC4 |
| Q9SIH0 | | 0.142 | 0.257 | 0.004 | 0.8 | 40S ribosomal protein S14-1 | RS141 |
| Q9SIM4 | | 0.302 | 0.495 | 0.015 | 0.7 | 60S ribosomal protein L14-1 | RL141 |
| Q93ZN2 | | 0.282 | 0.437 | 0.041 | 0.7 | Probable aldo-keto reductase 4 | ALKR4 |

| Q9LKR3 | | 1.119 | 1.835 | 0.041 | 0.7 | Mediator of RNA polymerase II  transcription subunit 37a | MD37A |
| --- | --- | --- | --- | --- | --- | --- | --- |
| Q9FMP3 | | 1.183 | 2.187 | 0.026 | 0.7 | Dihydropyrimidinase | DPYS |
| Q9S9N1 | | 1.651 | 2.616 | 0.004 | 0.7 | Heat shock 70 kDa protein 5 | HSP7E/BiP1 |
| P42798 | | 0.320 | 0.489 | 0.004 | 0.6 | 40S ribosomal protein S15a-1 | R15A1 |
| A8MS03 | | 0.126 | 0.199 | 0.026 | 0.6 | Ribosomal protein S6 | A8MS03 |
| A8MS28 | | 0.481 | 0.754 | 0.026 | 0.6 | Ribosomal L27e protein family | A8MS28 |
| Q9SEI3 | | 0.326 | 0.496 | 0.026 | 0.6 | 26S proteasome regulatory  subunit 10B homolog A | PS10A/RTP4A |
| Q9SII0 | | 0.257 | 0.395 | 0.009 | 0.6 | Probable histone H2A variant 2 | H2AV2 |
| Q39142 | | 2.316 | 3.472 | 0.041 | 0.6 | Chlorophyll a-b binding protein,  chloroplastic | Q39142 |
| P16181 | | 0.224 | 0.332 | 0.009 | 0.5 | 40S ribosomal protein S11-1 | RS111 |
| Q9SRV5 | | 2.348 | 3.690 | 0.041 | 0.5 | 5-  methyltetrahydropteroyltrigluta mate-homocysteine methyltransferase 2 | METE2 |
| P49107 | | 0.604 | 1.064 | 0.004 | 0.5 | Photosystem I reaction center  subunit N, chloroplastic | PSAN |
| P59259 | | 9.892 | 14.422 | 0.041 | 0.5 | Histone H4 | H4/HIS4 |
| Q9LHA8 | | 0.325 | 0.467 | 0.015 | 0.5 | Probable mediator of RNA  polymerase II transcription subunit 37c | MD37C |
| O04486 | | 0.251 | 0.354 | 0.041 | 0.5 | Ras-related protein RABA2a | RAA2A |
| P59233 | | 3.624 | 5.261 | 0.015 | 0.5 | Ubiquitin-40S ribosomal  protein S27a-3 | R27AC |
| Q8W4H7 | | 7.269 | 10.076 | 0.015 | 0.5 | Elongation factor 1-alpha 2 | EF1A2 |
| P52577 | | 2.145 | 3.732 | 0.002 | 0.5 | Isoflavone reductase homolog  P3 | IFRH |
| F4JWF7 | | 0.616 | 0.849 | 0.041 | 0.5 | DEAD/DEAH box RNA  helicase family protein | F4JWF7 |
| Q9SVR0 | | 0.114 | 0.183 | 0.009 | 0.5 | 60S ribosomal protein L13a-3 | R13A3 |
| P59224 | | 2.369 | 3.154 | 0.009 | 0.5 | 40S ribosomal protein S13-2 | RS132 |
| Q9SRZ6 | | 0.376 | 0.563 | 0.026 | 0.4 | Cytosolic isocitrate  dehydrogenase [NADP] | ICDHC/cICDH |
| Q9LZH9 | | 0.282 | 0.384 | 0.009 | 0.4 | 60S ribosomal protein L7a-2 | RL7A2 |
| Q9LD28 | | 0.620 | 1.017 | 0.041 | 0.4 | Histone H2A.6 | H2A6 |
| Q948K6 | | 0.232 | 0.315 | 0.015 | 0.4 | Ras-related protein RABG1 | RABG1 |
| P22953 | | 0.397 | 0.573 | 0.026 | 0.4 | Probable mediator of RNA polymerase II transcription  subunit 37e | MD37E |
| Q9SU58 | | 0.146 | 0.209 | 0.015 | 0.4 | ATPase 4, plasma membrane-  type | PMA4 |
| Q8H156 | | 1.697 | 2.498 | 0.009 | 0.4 | GTP-binding nuclear protein  Ran-3 | RAN3 |
| A0A1P8B2Y6 | | 0.197 | 0.287 | 0.026 | 0.4 | Plasma membrane ATPase | A0A1P8B2Y6 |
| A8MS75 | | 1.813 | 2.570 | 0.026 | 0.4 | Chlorophyll a-b binding protein,  chloroplastic | A8MS75 |
| Q9FJA6 | | 1.027 | 1.379 | 0.004 | 0.4 | 40S ribosomal protein S3-3 | RS33 |
| Q9FF90 | | 0.841 | 1.131 | 0.015 | 0.4 | 60S ribosomal protein L13-3 | RL133 |
| F4J3P1 | | 1.255 | 1.589 | 0.004 | 0.4 | Ribosomal protein L14p/L23e  family protein | F4J3P1 |
| Q9LXG1 | | 0.243 | 0.382 | 0.026 | 0.4 | 40S ribosomal protein S9-1 | RS91 |
| Q6ICZ8 | | 0.167 | 0.250 | 0.002 | 0.3 | Nascent polypeptide-associated complex subunit alpha-like  protein 3 | NACA3 |
| P0CJ47 | | 0.700 | 0.970 | 0.026 | 0.3 | Actin-3 | ACT3 |
| Q9SZ54 | | 0.346 | 0.476 | 0.041 | 0.3 | Putative glutathione peroxidase  7, chloroplastic | GPX7 |
| A0A1P8B767 | | 0.610 | 0.773 | 0.041 | 0.3 | Quinone reductase family  protein | A0A1P8B767 |
| Q8LB10 | | 0.117 | 0.141 | 0.015 | 0.3 | ATP-dependent Clp protease  proteolytic subunit-related protein 4, chloroplastic | CLPR4 |
| F4JJ94 | | 0.801 | 1.008 | 0.026 | 0.3 | General regulatory factor 1 | F4JJ94 |
| Q9LUD4 | | 0.326 | 0.414 | 0.041 | 0.3 | 60S ribosomal protein L18a-3 | R18A3 |
| Q93VH9 | | 2.069 | 2.492 | 0.015 | 0.2 | 40S ribosomal protein S4-1 | RS41 |
| O23254 | | 0.837 | 1.142 | 0.041 | 0.2 | Serine  hydroxymethyltransferase 4 | GLYC4 |
| O49299 | | 2.435 | 3.204 | 0.002 | 0.2 | Probable phosphoglucomutase,  cytoplasmic 1 | PGMC1/PGM1 |
|  | ***Proteins less abundant in P. cinnamomi inoculated samples compared to the control*** | | | | | | |

| F4J3Q8 | 0.345 | 0.099 | 0.004 | -3.5 | P-loop containing nucleoside triphosphate hydrolases  superfamily | F4J3Q8 |
| --- | --- | --- | --- | --- | --- | --- |
| P10795 | 0.878 | 1.543 | 0.818 | -3.1 | Ribulose bisphosphate carboxylase small chain 1A,  chloroplastic | RBS1A/RBCS1A |
| Q9FLN4 | 0.218 | 0.100 | 0.132 | -1.5 | 50S ribosomal protein L27,  chloroplastic | RK27 |
| Q9FZ47 | 1.255 | 0.641 | 0.065 | -1.3 | ACT domain-containing  protein ACR11 | ACR11 |
| O04603 | 0.412 | 0.201 | 0.041 | -1.2 | 50S ribosomal protein L5,  chloroplastic | RK5 |
| Q8RX32 | 0.513 | 0.260 | 0.026 | -0.6 | Tropinone reductase homolog  At1g07450 | TRNH2 |
| Q9SCW1 | 0.184 | 0.110 | 0.026 | -0.6 | Beta-galactosidase 1 | BGAL1 |
| F4JYM8 | 0.543 | 0.294 | 0.026 | -0.6 | Thiolase family protein | F4JYM8 |
| A0A1P8B485 | 0.402 | 0.257 | 0.015 | -0.6 | Protein translocase subunit  SecA | A0A1P8B485 |
| P25697 | 8.890 | 6.489 | 0.015 | -0.6 | Phosphoribulokinase,  chloroplastic | KPPR/PRK |
| Q9LRR9 | 1.487 | 0.662 | 0.002 | -0.6 | (S)-2-hydroxy-acid oxidase  GLO1 | GLO1/GOX1 |
| B3H4S6 | 0.434 | 0.300 | 0.041 | -0.3 | Dicarboxylate transporter 1 | B3H4S6 |
| P56778 | 19.522 | 15.325 | 0.002 | -0.3 | Photosystem II CP43 reaction  center protein | PSBC |
| P56761 | 14.189 | 11.445 | 0.015 | -0.3 | Photosystem II D2 | PSBD |
| Q9LF98 | 2.338 | 1.901 | 0.041 | -0.3 | Fructose-bisphosphate aldolase  8, cytosolic | ALFC8/FBA8 |
| F4KDZ4 | 2.902 | 1.688 | 0.026 | -0.3 | Malate dehydrogenase | F4KDZ4/PMDH2 |
| Q42525 | 0.553 | 0.340 | 0.026 | -0.3 | Hexokinase-1 | HXK1 |
| P27140 | 7.947 | 6.602 | 0.041 | -0.2 | Beta carbonic anhydrase 1,  chloroplastic | BCA1 |
| A0A1P8BG37 | 3.388 | 2.750 | 0.041 | -0.2 | Photosystem II stability/assembly factor,  chloroplast | A0A1P8BG37 |
| Q9SAU2 | 1.863 | 1.309 | 0.026 | -0.2 | D-ribulose-5-phosphate-3-  epimerase | Q9SAU2/RPE |

360 aUniProt accession, protein name and protein initials arise from the annotation using the
361 *Arabidopsis* proteome database as a reference. For more details on the abundance levels
362 per replicate consult S1 Table.
363 bData generated from SWATH-MS proteomics: median peak areas for 6 control cork oak
364 plants (Median C) and 6 plants inoculated with *P. cinnamomi* (Median I).
365 cFold change ratio logarithm of protein abundance in inoculated over control samples
366 greater than 1 or less than -1 (Log2FC).
367 dNon-parametric Mann Whitney U-test (MW) with statistical significance level set to less

368	than 5% (p<0.05).

369

## 370 Hierarchical clustering of differentially produced cork oak

371 **proteins**
372 The proteins with differential levels between inoculated and control samples were
373 clustered in a heatmap to allow better visualization of the protein variation patterns (Fig
374 4). Inoculated plants are clearly distinguished from control plants based on the profiles of
375 this protein dataset. In other words, eight months after a single inoculation of the cork oak
376 root with *P. cinnamomi*, the inoculated but asymptomatic plants revealed a leaf proteome
377 significantly different from the non-inoculated plants.

##### 379 Fig 4. Hierarchical clustering of differentially produced cork oak proteins. The heat

380 map clusters the expression patterns of the 80 proteins with altered abundances between
381 inoculated and control plants. Each column represents one cork oak plant; the control (C)
382 plants are the first 6 columns on the left, and the 6 columns on the right are the inoculated
383 (I) plants. The first 60 lines starting from the top of the heat map are proteins with an
384 increased level in the inoculated samples (red color code) and the 20 lines towards the
385 bottom, are proteins with a decreased level in the inoculated samples (green color code).
386 The color scale of the heat map ranges from -3 to 3 (from light green to red).
388 Two scenarios are possible for the inoculated plants: 1) the development of the
389 oomycete was restricted to the inoculation site, with no spread of the infection; or 2) the
390 development of the oomycete took place beyond the inoculation site, invading other
391 tissues, but the infection still did not affect the vegetative state of the host. For the first
392 hypothesis, the protein profiles observed in the leaves may be the result of the activation
393 of the systemic defence system, maintained in memory over time. But, for the second
394 hypothesis, the protein profiles of the inoculated plants may denote a homeostatic state
395 of continuous interaction with *P. cinnamomi*.
396 In this experiment, attempts were made to mimic field conditions,
397 which are hampered by limitations regarding the detection and quantification of the
398 oomycete. All the inoculated plants were used at the end of the experiment, and there was
399 no selection based on the re-isolation of the oomycete or the existence of infection
400 symptoms like leaf yellowing and wilting. Assessing cork oak decline in the field is based
401 on the degree of canopy defoliation, and even if *P. cinnamomi* is isolated from the roots
402 of declining trees, it is not possible to know the level or time of infection. Thus, evaluating
403 cork oak decline through the leaf immune response protein profile induced by *P.*
404 *cinnamomi* inoculation establishes a new approach for understanding the importance of
405 this oomycete to cork oak decline.

## 407 Association of proteins to GO functional categories and

408 **biological pathways**
409 Enrichment analysis was carried out on the selected dataset of 80 differential
410 proteins, which assigned several GO terms to the proteins (based on the *Arabidopsis*
411 *thaliana* proteins functional annotations), integrating them into the Gene Ontology
412 functional categories of “Biological Process” (GO_BP) or “Cellular Component”
413 (GO_CC). Within each classification, the significantly enriched terms (FDR<0.05; see
414 lists in S2 and S3 Tables) were assembled into groups of functionally related terms by
415 Cytoscape/ClueGO analysis and the most significantly enriched groups are presented in
416 Fig 5 and summarized in S4 and S5 Tables.
417 Thirty-six GO Biological Process terms were significantly enriched among the
418 differential proteins (S2 Table), which were grouped into 6 groups of functional related
419 GO terms (summarized in Fig 5a); the group *Pyridine-containing compound metabolic*
420 *process* has the highest enrichment score of all groups (12.9, corresponding to an FDR of
421 1.29 x 10^-4^), followed by *Monosaccharide catabolic process* (11.5), *Cellular metabolic*
422 *compound salvage* (10.7), *Protein-DNA complex assembly* (10.1), *Lipid oxidation* (10.0)
423 and *Response to endoplasmic reticulum stress* (10.0).
424 In the Cellular Component category, 15 GO terms were significantly enriched
425 among the differential proteins (S3 Table), which were grouped into 4 groups of
426 functional related GO terms (summarized in Fig 5b); the groups with the highest
427 enrichment scores belonged to *Cytosolic part* (82.1), *Stromule* (22.8), *Nucleosome* (14.8)
428 and *Photosystem* (14.1).

##### 430 Fig 5. Enrichment analysis applied to the subset of 80 differential proteins. The bars

431 represents the groups with higher enrichment score [−log2 (group FDR)] obtained for
432 each group of functional related GO terms; the enrichment of the Gene Ontology category
433 of GO_BP are showed in panel a), and those for GO_CC in panel b). Each group is labelled
434 by the most significant (<FDR) enriched term, used as representative of the total enriched
435 terms in each group that can be consulted in detail in Tables S2 and S3.
437 Furthermore, the enrichment analysis uncovered the most representative KEGG
438 or REACTOME biological pathways in this dataset of the 80 differential proteins, which
439 are listed in Table 4. Comparing the results from KEGG and REACTOME pathways, the
440 protein subset is enriched in *Ribosome* and *SRP-dependent cotranslational protein*
441 *targeting	to	membrane*,	with	the	highest	scores,	respectively,	and
442 *Glycolysis/Gluconeogenesis*	and	*Glucose	metabolism*	with	the	lowest	scores,
443 respectively.

##### 445 Table 4. Biological pathways uncovered for the selected dataset.

446

| **ID** | **Term** | **Source** | **Term FDR** | **Group FDR** | **Enrichment**  **score** | **Groups** | **% Associated**  **proteins** | **Number of**  **proteins** |
| --- | --- | --- | --- | --- | --- | --- | --- | --- |
| KEGG:03010 | Ribosome | KEGG | 6.46E-08 | 3.69E-08 | 24.7 | 4 | 5.49 | 20.0 |
| KEGG:00630 | Glyoxylate and dicarboxylate  metabolism | KEGG | 1.20E-03 | 6.86E-04 | 10.5 | 2 | 8.00 | 6.0 |
| KEGG:00710 | Carbon fixation in photosynthetic  organisms | KEGG | 4.03E-03 | 2.30E-03 | 8.8 | 3 | 7.25 | 5.0 |
| KEGG:00010 | Glycolysis / Gluconeogenesis | KEGG | 2.64E-02 | 1.51E-02 | 6.1 | 1 | 4.35 | 5.0 |
| R-ATH:1799339 | SRP-dependent cotranslational  protein targeting to membrane | REACTOME | 4.98E-10 | 3.98E-08 | 24.6 | 3 | 7.39 | 17.0 |
| R-ATH:3371497 | HSP90 chaperone cycle for  steroid hormone receptors (SHR) | REACTOME | 5.16E-05 | 1.06E-02 | 6.6 | 2 | 22.22 | 4.0 |
| R-ATH:70326 | Glucose metabolism | REACTOME | 2.99E-02 | 2.99E-02 | 5.1 | 1 | 4.76 | 3.0 |

449 The significantly enriched pathway with Reactome code R-ATH: 3371497-
450 *HSP90 chaperone cycle for steroid hormone receptors (SHR)* is very relevant in the
451 context of this investigation and of the available bibliography. The innate immunity and
452 plant defence in *Arabidopsis* are biological events that are associated with the biological
453 function of heat shock protein 90-2 as a molecular chaperone, involved in RPM1-
454 mediated resistance and component of the RPM1/RAR1/SGT1 complex [50]. To
455 circumvent the autoimmunity associated with high levels of immunity receptors, HSP90
456 proteins may assist in the formation of protein complexes that target the immune receptors
457 SNC1, RPS2, and RPS4 for degradation [51].

## 459 Protein abundance patterns associated with GO functional

460 **categories**

#### 461 GO biological process category

462 In the enrichment analysis, 18 proteins contributed significantly to certain
463 biological processes (GO_BP groups of enriched GO_BP terms), which are detailed in
464 Table 5. Of the six highlighted GO-BP groups, three stand out based on the constant
465 patterns of variation in the abundance of the associated proteins. These are: *Cellular*
466 *metabolic compound salvage* (GO group 3), with four down-accumulated proteins in the
467 inoculated plants; *Protein-DNA complex assembly* (GO group 5), with four up-accumulated
468 proteins in the inoculated plants and *Response to endoplasmic reticulum stress* (GO group
469 2), with two up-accumulated proteins in the inoculated plants. In the GO_BP groups
470 *Pyridine-containing compound metabolic process*, *Monosaccharide catabolic process*
471 and *Lipid oxidation*, proteins with different variation forms were housed in the same
472 group.

##### 474 Table 5: Proteins from the selected dataset assigned to Biological process GO terms

475 **with the highest enrichment scores.**
476

| **Enrichement analysis** | | **SWATH analysis** | | **Data analysis** | | | | |
| --- | --- | --- | --- | --- | --- | --- | --- | --- |
| **GO Group title and number** | **GO group^a^** | ***Arabidopsis***  **Uniprot**  **Accession^b^** | **Protein name (initials)^b^** | **Potential subcellular**  **location^c^** | **Potential pathway or biological**  **processes^d^** | **LOG2FC^e^** | | |
| Pyridine-containing compound metabolic process (4) | 4,6 | O04499 | 2,3-bisphosphoglycerate- independent phosphoglycerate mutase  1 (PMG1/iPGAM) | Cytoplasm | Glycolysis |  |  |  |
|  | 4 | Q9SRZ6 | Isocitrate dehydrogenase [NADP]  (ICDHC/cICDH) | Cytoplasm | Plant defense; Oxidative stress |  |  | |
|  | 4 | Q9LF98 | Fructose-bisphosphate aldolase 8  (ALFC8/FBA8) | Cytoplasm | Glycolysis; Stress signalling |  |  | |
| Monosaccharide catabolic process (6) | 6 | O49299 | Probable phosphoglucomutase, cytoplasmic 1  (PGMC1/PGM1) | Cytoplasm | Carbohydrate metabolism |  |  | |
|  | 4,6 | Q42525 | Hexokinase-1 (HXK1) | Cytoplasm  Nucleous | Glycolysis; Stress  signalling |  |  | |
|  | 4,6 | Q9SAU2 | D-ribulose-5-phosphate- 3-epimerase  (Q9SAU2/RPE) | Chloroplast | Photosynthesis |  |  | |
| Cellular metabolic compound salvage (3) | 3 | P10795 | Ribulose bisphosphate carboxylase small chain  1A (RBS1A/RBCS1A) | Chloroplast | Photorespiration; Photosynthesis |  |  | |
|  | 3 | P25697 | Phosphoribulokinase  (KPPR/PRK) | Chloroplast | Photosynthesis;  Plant defense |  |  | |
|  | 3 | Q9LRR9 | (S)-2-hydroxy-acid oxidase GLO1  (GLO1/GOX1) | Peroxisome | Plant defense; Photorespiration |  |  | |
|  | 3,1 | F4KDZ4 | Malate dehydrogenase  (F4KDZ4/PMDH2) | Peroxisome | Fatty acid ß-  oxidation |  |  | |
| Protein-DNA complex assembly (5) | 5 | A8MRL0 | Histone superfamily protein H3.3  (A8MRL0/AT4G40030) | Nucleus | DNA-binding; Protein  heterodimerization |  |  |  |
|  | 5,2 | Q9SEI3 | 26S proteasome regulatory subunit 10B homolog A  (PS10A/RTP4A) | Nucleus | Effector Triggered Imunity; ATPase activity |  |  |  |
|  | 5 | P59259 | Histone H4 (H4/HIS4) | Nucleus | Nucleosome assembly; Protein  heterodimerization |  |  |  |
|  | 5 | Q8LB10 | ATP-dependent Clp protease proteolytic subunit-related protein 4  (CLPR4) | Chloroplast | Plastid protein homeostasis; Protein degradation |  |  |  |
| Lipid oxidation (1) | 1 | P38418 | Lipoxygenase 2 (LOX2) | Chloroplast | Lipid metabolism;  Biotic stress |  |  |  |
|  | 1 | F4JYM8 | Thiolase family protein (F4JYM8/AACT1) | Peroxisome | Transferase activity |  |  | |
| Response to endoplasmic reticulum stress (2) | 2 | P27323 | Heat shock protein 90-1  (HSP901) | Cytoplasm | Chaperone; Plant  defense |  |  | |
|  | 2 | Q9S9N1 | Heat shock 70-5  (HSP7E/BiP1) | Endoplasmic  Reticulun | Chaperone; Plant  defense |  |  | |

477 aThe proteins included in more than one GO group were referenced only once in Table 5
478 with an indication of the numbers of the groups with which they were associated.
479 bThe table includes information about the protein names linked to *Arabidopis* Uniprot
480 Accessions, used as a reference for the cork oak leaf proteomic profiles.
481 cSuggestions of the potential subcellular locations, based on the annotation in protein
482 databases and available bibliography.
483 dInferences about the possible biological processes associated with proteins in the context
484 of this study.
485 eFold change ratio logarithm of protein abundance in inoculated samples over control
486 greater than 1 or less than -1 (Log2FC) shown as a colored bar. The numeric values are
487 presented in Table 3.
489 Within the proteins mapping to *Cellular metabolic compound salvage* (GO group
490 3), the protein Ribulose 1,5-bisphosphate carboxylase/oxygenase (RubisCO) small
491 subunit 1A (P10795; RBCS1A) was the one showing the most expressive negative
492 variation between inoculated and control samples (Log2FC=-3.1). Further, this protein
493 showed a very high coefficient of variation (% CV), both in control (151%) and
494 inoculated samples (152%). CV values may reflect the natural biological variability
495 observed for RubisCO in *Q. suber* and the corresponding RubisCO patchiness in the host's
496 response to the oomycete. In *Quercus ilex* inoculated with *P. cinnamomi* a decrease in the
497 abundance of RubisCO proteins was also found, which was correlated with the
498 tolerance/susceptibility of the provenances, being more accentuated in susceptible
499 provenances [52].
500 This protein is very abundant in plants and the amount of RubisCO in the leaves
501 is considered an indicator of the photosynthetic vigour and nitrogen availability. RBCS1A
502 is a member of the multigene family RBCS from *Arabidopsis* and Isumy and colleagues
503 [53] reported the additive effect of the expression of RBCS1A and RBCS3C genes on
504 Rubisco accumulation in *Arabidopsis* leaves. Therefore, low levels of RBCS1A in the *P.*
505 *cinnamomi* inoculated cork oak plants may indicate decreased levels of total RBCS
506 mRNA and a smaller content of RubisCO accumulated in the leaves. It will be interesting
507 to evaluate if the leaves of *P. cinnamomi* inoculated cork oak plants have a reduced
508 photosynthetic activity but, if this was a decrease, it appears not to have significantly
509 affected plant growth that was similar between inoculated and control plants.
510 Like	Rubisco,	the	chloroplastic	phosphoribulokinase	(P25697/PRK)	is
511 specifically associated with the Calvin-Benson cycle and catalyses D-ribulose 1,5-
512 bisphosphate formation, used by Rubisco with CO2 or O2 to form 3-phosphoglycerate (3-
513 PGA) and 2-phosphoglycolate (2-PG) [54, 55]. Similarly, this protein also showed lower
514 levels of accumulation in the *P. cinnamomi* inoculated plants in this study. The protein
515 D-ribulose-5-phosphate-3-epimerase protein (Q9SAU2/RPE) [56], mapping to GO
516 groups 4 and 6 and participating in the carbon photoassimilation cycle, also showed lower
517 levels of accumulation in the inoculated plants like RubisCO and PRK. It is likely that
518 the decrease in the accumulation of these proteins can compromise the levels of carbon
519 assimilated by the plant and the sequent synthesis of sugars, proteins, lipids and nucleic
520 acids. A similar effect was observed in *Q. ilex* inoculated with *P. cinnamomi* where many
521 proteins involved in the Calvin-Benson cycle, such as RubisCO large and small subunits,
522 phosphoglycerate	kinase,	glyceraldehyde-3-phosphate	dehydrogenase	B	and
523 transketolase 1 were also decreased [52]. The complementarity of these data makes sense
524 if	we	think	about	the	existence	of	macromolecular	complexes	formed	by
525 phosphoribulokinase and glyceraldehyde-3-phosphate dehydrogenase interacting with
526 the small peptide CP12, with relevance to the regulation of photosynthesis in the
527 chloroplasts [57, 58].
528 The two other proteins mapping to this GO group, (S)-2-hydroxy-acid oxidase,
529 with the alternative name of glycolate oxidase 1 (Q9LRR9; GOX1/GLO1) and malate
530 dehydrogenase (F4KDZ4; PMDH2), are known to be located in the peroxisomes, and
531 both showed decreased levels in the inoculated plants. GOX1 catalyses the conversion of
532 glycolate into glyoxylate with the production of H2O2 in the photorespiration pathway
533 (EC 1.1.3.15). Modulation of hydrogen peroxide accumulation has been suggested as the
534 mechanism adopted by the GOX protein family in *Arabidopsis* and *Nicotiana*
535 *benthamiana*, associated with PAMP-triggered immunity (PTI), host and nonhost defence
536 responses [59-61]. Furthermore, the defence pathways activated by different GOX genes
537 vary between plant species and depend on the type of interaction that occurs between
538 plants and pathogens or elicitors and GOX1-dependent defence responses may involve
539 salicylic acid (SA) and *WRKY62*-mediated pathways [59-61].
540 Concerning	malate	dehydrogenase	(F4KDZ4;	PMDH2),	in	2007,
541 Pracharoenwattana and colleagues [62] proposed a model in which the action of this
542 enzyme is the production of malate from oxaloacetate with NADH oxidation, recruited
543 for fatty acid ß-oxidation.
544 In summary, the decrease in the accumulation of these four proteins rebound on
545 photosynthesis and concomitant photorespiration, and may affect sugar metabolism; on
546 triacylglycerides oxidation as an alternative source of energy and gluconeogenic
547 intermediates; and in the activation of the defence system mediated by reactive oxygen
548 species (ROS) production and by changes in the redox state of the cells. Compromising
549 the photosynthetic efficiency in source tissues may result in a reduced supply of sugars
550 to sink tissues and less accumulation of soluble sugars.
551 Focusing on the *Pyridine-containing compound metabolic process* (GO group 4)
552 and *Monosaccharide catabolic process* (GO group 6), hexose sugars like
553 glucose are central molecules in plant metabolism and in sugar signaling. Cytosolic
554 resources of phosphate hexoses originating from starch mobilization and sucrose
555 hydrolysis are channeled for energy-producing and synthesis of biomolecule precursors
556 [63]. Proteins PGMC1/PGM1 (Probable phosphoglucomutase), PMG1/iPGAM1 (2,3-
557 bisphosphoglycerate-independent phosphoglycerate mutase 1), ALFC8/FBA8 (fructose-
558 bisphosphate aldolase 8) and HXK1 (Hexokinase 1) from GO groups 4 and 6 can be
559 grouped into two pairs according to their quantification pattern, but also to the role they
560 play in sugar metabolism and as sugar sensors. PGMC1/PGM1 is an enzyme that
561 participates in both the breakdown and synthesis of glucose (EC: 5.4.2.2.) and
562 PMG1/iPGAM1 is involved in the synthesis of pyruvate in glycolysis (EC: 5.4.2.12).
563 These proteins were more abundant in the inoculated samples of this study, revealing a
564 metabolic tendency in favor of energy production and reducing power as opposed to the
565 accumulation of sucrose and carbohydrates as reserve substances. The availability of
566 energy resulting from the functioning of enzymes may hamper energy-depending cell
567 actions, such as the movement of stomata, and this requirement was studied in
568 *Arabidopsis* through silencing the expression of glycolytic proteins. Silencing *iPGAM*
569 activity in *Arabidopsis* is associated with reduced stomatal function and plant phenotypes
570 with delayed development. This probably results from the decrease in ATP production
571 by the glycolytic pathway and also by tricarboxylic acid (TCA) cycle and oxidative
572 phosphorylation in consequence of the concomitant reduction in the levels of pyruvate
573 provided [64]. HXK1 and FBA8 were less abundant in the inoculated samples and both
574 enzymes are involved in glycolysis (EC: 2.7.1.1; EC: 4.1.2.13) and sucrose metabolism
575 [65], also being referred to as proteins involved in sugar and stress signaling [66, 67]. In
576 *Arabidopsis*, transcripts levels of *AtFBA8* showed increased expression after 24h of
577 glucose, fructose and sucrose treatment and these were responsive to ABA, SA, NaCl and
578 drought stresses [67]. A reduction in the production of these enzymes in the inoculated
579 cork oak plants may be a consequence of the imbalance of the metabolism towards the
580 production of energy. Knowing the subcellular location of HXK1 is essential to
581 understand the role it plays in response to biotic stresses, because HXK1 located in the
582 nucleus may interact with other proteins regulating the transcription of genes by binding
583 directly to the chromatin [68] and mitochondrial hexokinases can modulate programmed
584 cell death (PCD) [69].
585 Cytosolic ICDHC/cICDH (Isocitrate dehydrogenase [NADP]) protein, mapping
586 to GO group 4 and increasing its levels in inoculated plants, is potentially responsible for
587 2-oxoglutarate production for amino acid biosynthesis; however, in *Arabidopsis* cICDH
588 is not required for plant development and primary metabolism in optimal growth
589 conditions, instead, cICDH contributes to thiol–disulphide homeostasis during oxidative
590 stress [70]. The NADPH produced by cICDH may contribute to activate defence
591 responses to pathogen infection that are triggered by changes in cellular redox state [70].
592 Reprogramming gene expression in situations of biotic stress requires modulation
593 of transcriptional activity in the nucleus. Focusing now on *Protein-DNA complex*
594 *assembly* (GO group 5) and *Response to endoplasmic reticulum stress* (GO group 2),
595 several proteins were found with increased levels, including two histones.	It was
596 suggested that appropriate levels of H3.3 are required to avoid H1 deposition over gene
597 bodies preserving an adequate density of nucleosomes ideal for chromatin unfolding and
598 access to DNA methyltransferases that methylate gene bodies [71]. Besides, the local
599 enrichment	of	the	nuclear	histone	H3.3	variant	was	positively	correlated with
600 transcription of responsive genes [72, 73] and with gene body methylation [71]. Thus, the
601 higher levels of H3.3 protein in the inoculated plants in this study suggest an increased
602 access to DNA, allowing for modulation of transcription of biotic responsive-genes
603 through gene body methylation. Still related to the formation of histone-DNA tetrasome
604 is the *Arabidopis* chaperone NASP, described to bind to H3-H4 dimers and to stimulate
605 the conversion of dimers to tetramers, *in vitro* [74]. Furthermore, in tobacco and
606 *Arabidopsis* cell lines, the modifications observed in histone H3 in response to abiotic
607 stresses, with up-regulation of marker genes, happens together with histone H4
608 acetylation, revealing the parallel intervention of these histones [75]. Also, in the present study,
609 a significant up-accumulation of both H3.3 and H4 proteins were found in the leaves of
610 cork oak plants inoculated with *P. cinnamomi*.
611 To reach cellular homeostasis, protein degradation by proteolytic enzymes is a
612 regulated procedure used to adjust protein abundance and efficiency. This activity in the
613 chloroplasts requires Clp protease complexes composed by several protein catalytic
614 (ClpP3 to ClpP6) and non-catalytic (ClpR1 to ClpR4) subunits arranged	in ring-like
615 structures (P-ring and R-ring) in *Arabidopsis* surrounding the proteolytic chamber whose
616 activity is assisted by several chaperone members [for review see 76]. The assembly of
617 the rings of the Clp core complex is compromised if there is an uneven number of subunits
618 available for its formation. It was observed that reducing the abundance of the subunit
619 ClpP6 by 50% caused a reduction in the protein abundance of other P- and R-ring
620 components, interfering with the complex assembly and functionality [77]. Therefore, it
621 is reasonable to expect that the increase in accumulation of the ClpR4 subunit in the inoculated cork oak
622 samples, may point to the importance of protein degradation in the regulation
623 of the photosynthetic process mediated by the Clp complex. Additionally, knockdown of
624 protease subunits in tobacco allowed the identification of putative protease substrates
625 [77], including proteins involved in photosynthesis like PRK and RPE, which were found
626 to be down-accumulated in the cork oak samples inoculated with *P. cinnamomi* (Table

627	5).

628 Beyond the Clp complex, there are other biological mechanisms that predict
629 protein degradation, through proteasome complexes, to modulate the activity of disease
630 resistance proteins (R) in plant-pathogen interactions [78], and also other processes such
631 as the oxidative burst, hormone signaling, gene induction, and programmed cell death
632 [79]. In tobacco cells challenged by the elicitin cryptogein, the accumulation of 20S
633 proteasome subunits was observed simultaneously with the development of systemic
634 acquired resistance [80]. By analogy, the RPT4A protein highlighted in the leaf proteome
635 of the inoculated samples in the present study can be regarded as a defence-induced
636 subunit of 26S proteasome, with a possible role in plant defence reactions eventually
637 triggered by elicitins produced by *P. cinnamomi*. It is also possible to assume ATPase
638 activity for *Q. suber* RTP4A subunit based on the functional characterization of the 26S
639 proteasomal subunit RPT4a from *Solanum lycopersicum* that has an active ATPase site
640 and can modulate the resistance to the ToLCNDV virus by physically interacting with
641 viral DNA molecules [81].
642 Modifying the programming of host nuclear gene transcription in response to
643 biotic stress is one of the mechanisms adopted by oomycetes and promoted through
644 effector molecules. The target genes may be those that code for HSP (Heat Shock
645 Proteins) proteins, modulating the role played by these molecular chaperones; these are
646 active partners of numerous enzyme complexes and are responsible for the folding and
647 unfolding of proteins included in protein degradation/renaturation and movement of
648 signaling proteins and transcription factors into cell organelles [82, 83]. In *Q. suber* leaves
649 inoculated with *P. cinnamomi*, the accumulation of HSP70-5 (Q9S9N1) and HSP90-1
650 (P27323) proteins was higher than in control samples. Knowing the activities of these
651 proteins in other plant species, it can be inferred the possible role they may play in the
652 interaction between	cork oak and *P. cinnamomi*. Song et al	(2015) reported	the
653 identification of a *P. sojae* intracellular CRN (Crinkler or crinkling- and necrosis-
654 inducing protein) effector which directly interacts with promoters of the genes encoding
655 HSP proteins, preventing the binding of specific transcription factors [84]. The expression
656 of the defence-related genes in *Arabidopsis*, *N. benthamiana* and soybean is then
657 changed, unbalancing the host's resistance level to *Phytophthora* species [84]. More
658 recently, HSP70s have been noted as proteins that interact with RXLR effectors produced
659 by *P. infestans* and that get involved in *N. benthamiana* defence response by stimulating
660 programmed cell death mediated by MAPK signaling and suppressing the growth of the
661 pathogen [85]. It was also reported the importance of protein complexes formed between
662 HSP90s and co-chaperones in the activation of defence mechanisms mediated by
663 resistance (R) proteins after the detection of pathogen effector molecules [86]. Preventing
664 the formation of these protein complexes by inhibiting the binding of HSP90 has
665 implications for the accumulation of R proteins and resistance mediated by these proteins
666 [86]. In *Arabidopsis*, the cytosolic AtHSP90.1 was the only HSP90 isoform significantly
667 induced after inoculation with *Pseudomonas* (*Pst*) strains containing avirulence genes
668 (*avrRpm1* and *avrRpt2*), and was required for the full resistance mediated by one of the
669 corresponding R proteins [87]. It is then expected that the host's resistance proteins will
670 recognize the effector molecules secreted by *P. cinnamomi* and activate the defence
671 responses with the collaboration of chaperones and co-chaperones.
672 The disclosed *Q. suber* HSP70-5 is recognized as a homolog of the *Arabidopsis*
673 BIP1 for Binding Immunoglobulin Protein or Binding Protein (BiP), a chaperone set in
674 the endoplasmic reticulum (ER) lumen, known to bind a membrane-associated
675 transcription factor (TF) under non-stressed conditions [88]. The interaction between BiP1
676 and the TF is the requirement to turn on or off a protein secretory signaling pathway via
677 ER-Golgi-Nucleus, ending with the transcription of stress response genes [88]. In
678 soybean, the BiP protein was described as a negative regulator of a stress-induced cell
679 death response [89] and, in *Arabidopsis*, Wang et al. (2005) reported the implications of
680 *BiP 2* silencing on the secretion of pathogenesis related-proteins, compromising the
681 systemic acquired resistance against bacterial pathogens [90].
683 When looking at the enrichment of *Lipid oxidation* (GO group 1), two differential
684 proteins were assigned to it, lipoxygenase 2 (LOX2; P38418) and thiolase family protein
685 (AACT1; F4JYM8), although with different patterns of variation: LOX2 was more
686 abundant in the inoculated plants and AACT1 was less abundant. By homology to the
687 Arabidopsis ACCT1 isoform, it is expected for *Q. suber* ACT1 to be located in the
688 peroxisome, based on the presence of two alternative targeting sequences PTS1 and PTS2
689 motives found in AtACCT1, excluding a metabolic function related to isoprenoid
690 biosynthesis [91, 92]. Jin et al (2012) found a strong expression of *AtACCT1* in the
691 vascular system of the aerial organs and roots of *Arabidioposis*, verifying that gene silencing or induction
692 of abiotic stresses did not result in an evident phenotypic response [93]. In *Q. suber*
693 inoculated by *P. cinnamomi*, the leaf proteome reveals a reduction in the production of a
694 thiolase AACT1 protein, apparently included in its defence strategy.
695 The involvement of LOX2 in the cork oak defence response may be associated
696 with the production of jasmonic acid (JA) via the Vick and Zimmerman pathway [94].
697 Recently, it was confirmed that LOX2 forms a protein complex with AOS (allene oxide
698 synthase) and AOC2 (allene oxide cyclase), two proteins that also participate in JA
699 precursor biosynthesis, located in the inner envelope of the *Arabidopsis* chloroplasts [95].
700 The formation of this molecular complex is evident in the effectiveness of JA production
701 to the disadvantage of other products resulting from parallel reactions during oxylipin
702 biosynthesis, guiding the defence response to the activation of genes responsive to JA
703 [95]. Sometimes, depending on the needs of the pathogen, the signaling reactions are a
704 balance between the activation of the salicylic acid (SA) pathway with suppression of the
705 jasmonic acid (JA) pathway or vice versa [96]. Nevertheless, Starý et al. (2019) conclude
706 that the level of resistance induced in different tomato genotypes after β-cryptogein
707 treatment correlated with the upregulation of defence genes and activated ethylene and
708 JA signaling but not SA signaling [97]. Other authors refer to a biphasic defence response
709 in avocado against the hemibiotroph *P. cinnamomi*, which initially involves SA-mediated
710 gene expression followed by the enrichment of JA-mediated defence from 18 to 24 hours
711 post-inoculation [37].
712 Finally, when analysing the GO Cellular Component category, the highest
713 enrichment scores were obtained for *Cytosolic part* (82.1), *Stromule* (22.8), *Nucleosome*
714 (14.8) and *Photosystem* (14.1). In a simplified view, it is recognized that achieving new
715 cell balances during interaction with pathogenic organisms requires the remodeling of
716 physiological processes by the action of cytoplasmic or organelle-associated enzymes and
717 the modulation of transcription factors for nuclear gene expression, which are energy-
718 dependent processes. Three of the obtained categories fit this profile with the exception
719 of *Stromule*, which is a novelty for the host-*P. cinnamomi* interactions. Nevertheless,
720 there were previous descriptions for the importance of the communication between
721 cellular organelles during immune responses carried out through stroma-filled tubular
722 structures (stromules) of the chloroplasts-to-nucleus, which use them as a support for the
723 exchange of molecules integrated into the defence response [98, 99]. Caplan et al (2015)
724 observed the induction of stromules in response to viral and bacterial effectors after
725 recognition by host receptors (ETI; effector-triggered immunity) [98]. In plants infected
726 with tobacco mosaic virus (TMV) a hypersensitive response is observed at the infection
727 site and in the border regions with increased production of stromules in both areas,
728 probably stimulated by the production of pro-defence signaling molecules like H2O2, O2-
729 and SA [99]. During innate immunity, the cellular relocation of chloroplasts in the nucleus
730 surroundings is dependent on the organization of microtubules ~~(MT)~~ in connection with
731 the anchoring points provided by actin filaments ~~(AF)~~ to enhance the effectiveness of the
732 communication between these organelles [100]. In 2013, Sghaier-Hammami et al.
733 reported the up-accumulation of actin in holm oak plants inoculated with *P. cinnamomi*
734 [52]. The published information reinforces the importance of the *Stromules* GO category
735 highlighted in the present study for the cork oak-*P. cinnamomi* interaction. Moreover, the cork
736 oak leaf proteome data suggests a possible function for stromules in long-term defence
737 responses, far from the inoculation point, in close connection with the production and
738 transport of signaling molecules.

# 740 Conclusions

741 In this work, the proteomes of cork oak plants submitted to biotic stress-induced
742 by *P. cinnamomi* inoculation are revealed for the first time. Among the 424 proteins
743 confidently quantified in the inoculated and non-inoculated plants, a dataset of 80 proteins
744 was selected based on the abundance variability observed between the experimental
745 conditions. The immune response of the plants was analysed eight months after the
746 inoculation event, and, at that moment, there were no evident phenotypic differences
747 between inoculated and non-inoculated plants. Nevertheless, the hierarchical clustering
748 of differentially produced cork oak proteins shows two different groups of plants,
749 matching to the experimental conditions. By comparing protein profiles, it was observed
750 that the number of proteins in which the abundance increased in the inoculated plants is
751 3 times greater than the number of proteins in which there was a decrease in abundance.
752 Therefore, the defence responses induced in the host by *P. cinnamomi* in the long term
753 and distant from the inoculation site are inscribed in the proteome of the leaves,
754 reproducing the in progress homeostatic state of the plants. The results obtained in this
755 study increase the possibilities of screening trees infected with *P. cinnamomi* using
756 protein markers identified in the leaves without the need to isolate the oomycete from the
757 roots of the host or surrounding soil.
758 The homeostatic state of the inoculated cork oak plants was characterized by
759 protein patterns associated with differential biological processes occurring potentially in
760 different subcellular organelles. When performing the enrichment analysis, eighteen
761 proteins were highlighted, and their possible functions in an immune response context
762 were discussed. In short, the decrease in the accumulation of photosynthesis enzymes and
763 concomitant photorespiration may compromise the levels of carbon assimilated by the
764 plant and its development, although throughout the experiment, no differences in growth
765 were observed. The dynamics of proteins associated with sugar metabolism and sugar
766 signaling reveals a metabolic tendency *in favor* of energy production and reducing power
767 as opposed to the accumulation of sucrose and carbohydrates as reserve substances.
768 The reprogramming of gene expression, eventually in response to the action of
769 effector molecules produced by *P. cinnamomi* is a major function associated with proteins
770 that are in greater abundance in the inoculated plants. It is also clear the participation of
771 proteolytic complexes and chaperones in the cork oak immune response and of proteins
772 sensitive to changes in the redox state of the cell promoted by ROS species.
773 In addition, the cork oak leaf proteome data suggests the importance of the
774 communication between cellular organelles mediated by stromules in the long-term
775 defence responses. Immune response amplification and effectiveness may be dependent
776 on the repositioning of the chloroplasts close to the nucleus and the transfer of pro-
777 defence molecules such as SA, JA and H2O2.

# 780 References

781 1. Serrano MS, Rios P, Gonzalez M, Sanchez ME. Experimental minimum threshold 782	for *Phytophthora cinnamomi* root disease expression on *Quercus suber*. Phytopathol 783	Mediterr. 2015:461-4.

784	2. Camilo-Alves CDEP, da Clara MIE, Ribeiro NMCD. Decline of Mediterranean 785	oak trees and its association with *Phytophthora cinnamomi*: a review. European 786	Journal of Forest Research. 2013;132(3):411-32. doi: 10.1007/s10342-013-0688-z. 787	PubMed PMID: WOS:000317422900002.

788	3. Vessella F, López-Tirado J, Simeone MC, Schirone B, Hidalgo PJ. A tree species 789	range in the face of climate change: cork oak as a study case for the Mediterranean 790	biome. Eur J For Res. 2017;136(3):555-69.

791	4. Camilo-Alves CS, Vaz M, Da Clara MIE, Ribeiro NMDA. Chronic cork oak

792	decline and water status: new insights. New For. 2017;48(6):753-72.

793	5. Avila J, Linares J, García-Nogales A, Sánchez M, Gómez-Aparicio L. Across- 794	scale patterning of plant–soil–pathogen interactions in *Quercus suber* decline. Eur J 795	For Res. 2017;136(4):677-88.

796	6. Mendes MP, Ribeiro L, David TS, Costa A. How dependent are cork oak 797	(*Quercus suber L.*) woodlands on groundwater? A case study in southwestern 798	Portugal. Forest Ecol Manag. 2016;378:122-30.

799	7. Tiberi R, Branco M, Bracalini M, Croci F, Panzavolta T. Cork oak pests: a review

800	of insect damage and management. Ann For Sci. 2016;73(2):219-32.

801	8. Henriques J, Barrento MJ, Bonifácio L, Gomes AA, Lima A, Sousa E. Factors 802	affecting the dispersion of *Biscogniauxia mediterranea* in Portuguese cork oak 803	stands. Silva Lusit. 2014;22(1):83-97.

804	9. Moricca S, Linaldeddu BT, Ginetti B, Scanu B, Franceschini A, Ragazzi A. 805	Endemic and emerging pathogens threatening cork oak trees: management options 806	for conserving a unique forest ecosystem. Plant Dis. 2016;100(11):2184-93.

807	10. Arosa ML, Bastos R, Cabral JA, Freitas H, Costa SR, Santos M. Long-term 808	sustainability of cork oak agro-forests in the Iberian Peninsula: model-based 809	approach aimed at supporting the best management options for the montado 810	conservation. Ecol Modell. 2017;343:68-79.

811	11. Horta M, Caetano P, Medeira C, Maia I, Cravador A. Involvement of the β-	812	cinnamomin elicitin in infection and colonisation of cork oak roots by *Phytophthora* 813	*cinnamomi*. Eur J Plant Pathol. 2010;127(3):427-36.

814	12. Horta M, Sousa N, Coelho AC, Neves D, Cravador A. *In vitro* and *in vivo*	815	quantification of elicitin expression in *Phytophthora cinnamomi*. Physiol Mol Plant 816	Pathol. 2008;73(1-3):48-57.

817	13. Islam M, Hussain H, Russo R, Chambery A, Amoresano A, Schallmey A, et al. 818	Functional analysis of elicitins and identification	of cell	wall	proteins	in 819	*Phytophthora cinnamomi*. Physiol Mol Plant Pathol. 2019;107:21-32.

820	14. Hardham AR, Blackman LM. *Phytophthora cinnamomi*. Mol Plant Pathol.

821	2018;19(2):260-85.

822	15. Meyer FE, Shuey LS, Naidoo S, Mamni T, Berger DK, Myburg AA, et al. Dual 823	RNA-sequencing of *Eucalyptus nitens* during *Phytophthora cinnamomi* challenge 824	reveals pathogen and host factors influencing compatibility. Front Plant Sci. 825	2016;7:191.

826	16. Kale S, Gu B, Capelluto D, Dou D, Feldman E, Rumore A, et al. External	827	phosphatidylinositol-3-phosphate mediates host cell entry by eukaryotic pathogen 828	effectors. Cell. 2010;142(2):284-95.

829	17. Kale SD, Tyler BM. Entry of oomycete and fungal effectors into plant and animal

830	host cells. Cell Microbiol. 2011;13(12):1839-48.

831	18. Wang H, Ren Y, Zhou J, Du J, Hou J, Jiang R, et al. The cell death triggered by 832	the nuclear localized RxLR effector PITG_22798 from *Phytophthora infestans* is 833	suppressed by the effector AVR3b. Int J Mol Sci. 2017;18(2):409.

834	19. Gascuel Q, Buendia L, Pecrix Y, Blanchet N, Muños S, Vear F, et al. RXLR and 835	CRN effectors from the sunflower downy mildew pathogen *Plasmopara halstedii* 836	induce hypersensitive-like responses in resistant sunflower lines. Front Plant Sci. 837	2016;7:1887.

838	20. Kong L, Qiu X, Kang J, Wang Y, Chen H, Huang J, et al. A *Phytophthora* effector 839	manipulates host histone acetylation and reprograms defence gene expression to 840	promote infection. Current Biol. 2017;27(7):981-91.

841	21. Jones J, Dangl. J. The plant immune system. Nature. 2006;444:323-9.

842	22. Hardoim P, Guerra R, Rosa da Costa A, Serrano M, Sánchez M, Coelho A. 843	Temporal metabolic profiling of the *Quercus suber–Phytophthora cinnamomi* 844	system by middle‐ infrared spectroscopy. For Pathol. 2016;46(2):122-33.

845	23. Engelbrecht J, Van den Berg N. Expression of defence-related genes against	846	*Phytophthora cinnamomi* in five avocado rootstocks. S Afr J Sci. 2013;109(11-12):1- 847	8.

848	24. Serrazina S, Santos C, Machado H, Pesquita C, Vicentini R, Pais MS, et al. 849	Castanea root transcriptome in response to *Phytophthora cinnamomi* challenge. Tree 850	Genet Genomes. 2015;11(1):6.

851	25. Coelho AC, Horta M, Ebadzad G, Cravador A. *Quercus suber–P. cinnamomi* 852	interaction: hypothetical molecular mechanism model. NZ	J	Forestry	Sci. 853	2011;(41S):143-57.

854	26. Flor HH. Current status of the gene-for-gene concept. Annu Rev Phytopathol.

855	1971;9(1):275-96.

856	27. van der Hoorn RA, Kamoun S. From guard to decoy: a new model for perception

857	of plant pathogen effectors. Plant Cell. 2008;20(8):2009-17.

858	28. Santos C, Duarte S, Tedesco S, Fevereiro P, Costa RL. Expression profiling of 859	*Castanea* genes during resistant and susceptible interactions with the oomycete 860	pathogen *Phytophthora cinnamomi* reveal possible mechanisms of immunity. Front 861	Plant Sci. 2017;8:515.

862 29. Dodds PN, Rathjen JP. Plant immunity: towards an integrated view of plant-
863 pathogen interactions. Nat Rev Genet. 2010;11(8):53948.
864 30. Fu ZQ, Dong X. Systemic acquired resistance: turning local infection into global
865 defence. Annu Rev Plant Biol. 2013;64:839-63.
866 31. Gao Q-M, Zhu S, Kachroo P, Kachroo A. Signal regulators of systemic acquired
867 resistance. Front Plant Sci. 2015;6:228.
868 32. Tada Y, Spoel SH, Pajerowska-Mukhtar K, Mou Z, Song J, Wang C, et al. Plant 869	immunity requires conformational changes of NPR1 via	S-nitrosylation	and 870	thioredoxins. Science. 2008;321(5891):952-6. Epub	2008/07/19. doi: 871	10.1126/science.1156970. PubMed PMID: 18635760; PubMed Central PMCID: 872	PMCPMC3833675.

873	33. Zhou J-M, Trifa Y, Silva H, Pontier D, Lam E, Shah J, et al. NPR1 differentially 874	interacts with members of the TGA/OBF family of transcription factors that bind an 875	element of the PR-1 gene required for induction by salicylic acid. Mol Plant Microbe 876	Interact. 2000;13(2):191-202.

877	34. Liu T, Song T, Zhang X, Yuan H, Su L, Li W, et al. Unconventionally secreted 878	effectors of two filamentous pathogens target plant salicylate biosynthesis. Nat	879	Commun. 2014;5(1):1-10.

| 880 | 35. Chang YH, Yan HZ, Liou RF. A novel elicitor protein from *Phytophthora* |
| --- | --- |
| 881 | *parasitica* induces plant basal immunity and systemic acquired resistance. Mol Plant |
| 882 | Pathol. 2015;16(2):123-36. |
| 883 | 36. Oßwald W, Fleischmann F, Rigling D, Coelho A, Cravador A, Diez J, et al. |
| 884 | Strategies of attack and defence in woody plant–*Phytophthora* interactions. For |
| 885 | Pathol. 2014;44(3):169-90. |
| 886 | 37. van den Berg N, Mahomed W, Olivier NA, Swart V, Crampton BG. |
| 887 | Transcriptome analysis of an incompatible *Persea americana-Phytophthora* |
| 888 | *cinnamomi* interaction reveals the involvement of SA-and JA-pathways in a |
| 889 | successful defence response. PloS One. 2018;13(10):e0205705. |
| 890 | 38. van den Berg N, Christie J, Aveling T, Engelbrecht J. Callose and β‐ 1, 3‐ |
| 891 | glucanase inhibit *Phytophthora cinnamomi* in a resistant avocado rootstock. Plant |
| 892 | Pathol. 2018;67(5):1150-60. |
| 893 | 39. Aizat WM, Ibrahim S, Rahnamaie-Tajadod R, Loke K-K, Goh H-H, Noor NM. |
| 894 | Proteomics (SWATH-MS) informed by transcriptomics approach of tropical herb |
| 895 | *Persicaria minor l*eaves upon methyl jasmonate elicitation. PeerJ. 2018;6:e5525. |
| 896 | 40. Bradford MM. Rapid and sensitive method for quantitation of microgram |
| 897 | quantities of protein utilizing principle of protein-dye binding. Anal Biochem. |
| 898 | 1976;72(1-2):248-54. doi: DOI 10.1006/abio.1976.9999. PubMed PMID: |
| 899 | WOS:A1976BU74400029. |
| 900 | 41. Anjo SI, Santa C, Manadas B. Short GeLC‐ SWATH: A fast and reliable |
| 901 | quantitative approach for proteomic screenings. Proteomics. 2015;15(4):757-62. |
| 902 | 42. Vizcaíno JA, Csordas A, Del-Toro N, Dianes JA, Griss J, Lavidas I, et al. 2016 |
| 903 | update of the PRIDE database and its related tools. Nucleic Acids Res. |
| 904 | 2016;44(D1):D447-D56. |
| 905 | 43. Pinto PI, Andrade AR, Moreira C, Zapater C, Thorne MA, Santos S, et al. |
| 906 | Genistein and estradiol have common and specific impacts on the sea bass |
| 907 | (*Dicentrarchus labrax*) skin-scale barrier. J Steroid Biochem Mol Biol. |
| 908 | 2019;195:105448. |
| 909 | 44. Pinto PI, Andrade AR, Thorne MA, Estêvão MD, Canario AV, Power DM. |
| 910 | Estradiol and genistein effects on the sea bass *(Dicentrarchus labrax*) scales: |
| 911 | Transcriptome dataset. Data Brief. 2019;27:104587. |
| 912 | 45. Isserlin R, Merico D, Voisin V, Bader GD. Enrichment Map–a Cytoscape app to |
| 913 | visualize and explore OMICs pathway enrichment results. F1000 Res. 2014;3. |
| 914 | 46. Bindea G, Mlecnik B, Hackl H, Charoentong P, Tosolini M, Kirilovsky A, et al. |
| 915 | ClueGO: a Cytoscape plug-in to decipher functionally grouped gene ontology and |

916	pathway annotation networks. Bioinformatics. 2009;25(8):1091-3. doi:

917	10.1093/bioinformatics/btp101. PubMed PMID: WOS:000265094400021.

918	47. de Hoon MJL, Imoto S, Nolan J, Miyano S. Open source clustering software.

919	Bioinformatics. 2004;20(9):1453-4. doi: 10.1093/bioinformatics/bth078.

920	48. Marcelino AC. Aspetos da interação entre *Phytophthora cinnamomi* e a doença

921	do declínio em *Q. suber* e *Q. rotundifolia*: University of Algarve; 2001.

922	49. Coelho AC, Lima M, Neves D, Cravador A. Genetic diversity of two evergreen 923	oaks [*Quercus suber* (L.) and *Quercus ilex* subsp. *rotundifolia* (Lam.)] in Portugal 924	using AFLP markers. Silvae Genet. 2006;55(1-6):105-18.

| 925 | 50. Hubert DA, He Y, McNulty BC, Tornero P, Dangl JL. Specific *Arabidopsis* |
| --- | --- |
| 926 | HSP90. 2 alleles recapitulate RAR1 cochaperone function in plant NB-LRR disease |
| 927 | resistance protein regulation. Proc Natl Acad Sci U S A. 2009;106(24):9556-63. |

| 928 | 51. Huang S, Monaghan J, Zhong X, Lin L, Sun T, Dong OX, et al. HSP 90s are |
| --- | --- |
| 929 | required for NLR immune receptor accumulation in *Arabidopsis*. Plant J. |
| 930 | 2014;79(3):427-39. |
| 931 | 52. Sghaier-Hammami B, Valero-Galvàn J, Romero-Rodríguez MC, Navarro-Cerrillo |
| 932 | RM, Abdelly C, Jorrín-Novo J. Physiological and proteomics analyses of Holm oak |
| 933 | (*Quercus ilex* subsp. *ballota* [Desf.] Samp.) responses to *Phytophthora cinnamomi*. |
| 934 | Plant Physiol Biochem. 2013;71:191-202. |
| 935 | 53. Izumi M, Tsunoda H, Suzuki Y, Makino A, Ishida H. RBCS1A and RBCS3B, two |
| 936 | major members within the *Arabidopsis* RBCS multigene family, function to yield |
| 937 | sufficient Rubisco content for leaf photosynthetic capacity. J Exp Bot. |
| 938 | 2012;63(5):2159-70. |
| 939 | 54. Kleffmann T, Russenberger D, von Zychlinski A, Christopher W, Sjölander K, |
| 940 | Gruissem W, et al. The *Arabidopsis thaliana* chloroplast proteome reveals pathway |
| 941 | abundance and novel protein functions. Curr Biol. 2004;14(5):354-62. |

942	55. Maurino VG, Engqvist MK. 2-Hydroxy acids in plant metabolism. The

943	Arabidopsis book: American Society of Plant Biologists; 2015.

944	56. Goulas E, Schubert M, Kieselbach T, Kleczkowski LA, Gardeström P, Schröder 945	W, et al. The chloroplast lumen and stromal proteomes of *Arabidopsis thaliana* show 946	differential sensitivity to short‐ and long‐ term exposure to low temperature. Plant 947	J. 2006;47(5):720-34.

948	57. Marri L, Sparla F, Pupillo P, Trost P. Co-ordinated gene expression of 949	photosynthetic glyceraldehyde-3-phosphate dehydrogenase, phosphoribulokinase, 950	and CP12 in *Arabidopsis thaliana*. J Exp Bot. 2005;56(409):73-80.

951	58. Graciet E, Lebreton S, Gontero B. Emergence of new regulatory mechanisms in 952	the Benson–Calvin pathway via protein–protein interactions: a glyceraldehyde‐ 3‐ 953	phosphate	dehydrogenase/CP12/phosphoribulokinase	complex.	J	Exp	Bot. 954	2004;55(400):1245-54.

955	59. Rojas CM, Senthil-Kumar M, Wang K, Ryu CM, Kaundal A, Mysore KS. 956	Glycolate oxidase modulates reactive oxygen species-mediated signal transduction 957	during nonhost resistance in *Nicotiana benthamiana* and *Arabidopsis*. Plant Cell. 958	2012;24(1):336-52. Epub 2012/01/31. doi: 10.1105/tpc.111.093245. PubMed PMID:

959	22286136; PubMed Central PMCID: PMCPMC3289552.

960	60. Rojas CM, Mysore KS. Glycolate oxidase is an alternative source for H2O2 961	production during plant defence responses and functions independently from 962	NADPH oxidase. Plant Signal Behav. 2012;7(7):752-5.

963	61. Xu Y-P, Yang J, Cai X-Z. Glycolate oxidase gene family in *Nicotiana* 964	*benthamiana*: genome-wide identification and functional analyses in disease 965	resistance. Sci Rep. 2018;8(1):1-11.

966	62. Pracharoenwattana I, Cornah JE, Smith SM. *Arabidopsis* peroxisomal malate 967	dehydrogenase functions in β‐ oxidation but not in the glyoxylate cycle. Plant J. 968	2007;50(3):381-90.

969	63. Everard J, Loescher W. Primary Products of Photosynthesis, Sucrose and Other 970	Soluble Carbohydrates. In: Thomas B, Murray BG, Murphy	DJ, editors. 971	Encyclopedia of Applied Plant Sciences. 1: Waltmam, MA: Academic Press; 2017. 972	p. 96-104.

973	64. Zhao Z, Assmann SM. The glycolytic enzyme, phosphoglycerate mutase, has 974	critical roles in stomatal movement, vegetative growth, and pollen production in 975	*Arabidopsis thaliana*. J Exp Bot. 2011;62(14):5179-89.

976	65. Gross W, Lenze D, Nowitzki U, Weiske J, Schnarrenberger C. Characterization, 977	cloning, and evolutionary history of the chloroplast and cytosolic class I aldolases of 978	the red alga *Galdieria sulphuraria*. Gene. 1999;230(1):7-14.

979	66. Rosa M, Prado C, Podazza G, Interdonato R, González JA, Hilal M, et al. Soluble 980	sugars: Metabolism, sensing and abiotic stress: A complex network in the life of 981	plants. Plant Signal Behav. 2009;4(5):388-93.

982	67. Lu W, Tang X, Huo Y, Xu R, Qi S, Huang J, et al. Identification and 983	characterization of fructose 1, 6-bisphosphate aldolase genes in *Arabidopsis* reveal a 984	gene family with diverse responses to abiotic stresses. Gene. 2012;503(1):65-74.

985	68. Cho Y-H, Yoo S-D, Sheen J. Regulatory functions of nuclear hexokinase1

986	complex in glucose signaling. Cell. 2006;127(3):579-89.

987	69. Kim M, Lim J-H, Ahn CS, Park K, Kim GT, Kim WT, et al. Mitochondria- 988	associated hexokinases play a role in the control of programmed cell death in 989	*Nicotiana benthamiana*. Plant Cell. 2006;18(9):2341-55.

990	70. Mhamdi A, Mauve C, Gouia H, Saindrenan P, Hodges M, Noctor G. Cytosolic 991	NADP‐ dependent isocitrate dehydrogenase contributes to redox homeostasis and 992	the regulation of pathogen responses in *Arabidopsis* leaves. Plant Cell Environ. 993	2010;33(7):1112-23.

994	71. Wollmann H, Stroud H, Yelagandula R, Tarutani Y, Jiang D, Jing L, et al. The 995	histone H3 variant H3. 3 regulates gene body DNA methylation in *Arabidopsis* 996	*thaliana*. Genome Biol 2017;18(1):94.

997	72. Nie X, Wang H, Li J, Holec S, Berger F. The HIRA complex that deposits the

| 998  999 | histone H3. 3 is conserved in *Arabidopsis* and facilitates transcriptional dynamics.  Biol Open. 2014;3(9):794-802. |
| --- | --- |
| 1000 | 73. Wollmann H, Holec S, Alden K, Clarke ND, Jacques P-E, Berger F. Dynamic |
| 1001 | deposition of histone variant H3. 3 accompanies developmental remodeling of the |
| 1002 | *Arabidopsis* transcriptome. PLoS Genet. 2012;8(5):e1002658. |
| 1003 | 74. Maksimov V, Nakamura M, Wildhaber T, Nanni P, Ramström M, Bergquist J, et |
| 1004 | al. The H3 chaperone function of NASP is conserved in *Arabidopsis*. Plant J. |
| 1005 | 2016;88(3):425-36. |
| 1006 | 75. Sokol A, Kwiatkowska A, Jerzmanowski A, Prymakowska-Bosak M. Up- |
| 1007 | regulation of stress-inducible genes in tobacco and *Arabidopsis* cells in response to |
| 1008 | abiotic stresses and ABA treatment correlates with dynamic changes in histone H3 |
| 1009 | and H4 modifications. Planta. 2007;227(1):245-54. |
| 1010  1011 | 76. Clarke AK. The chloroplast ATP‐ dependent Clp protease in vascular plants–new dimensions and future challenges. Physiol Plant. 2012;145(1):235-44. |
| 1012 | 77. Moreno JC, Martínez-Jaime S, Schwartzmann J, Karcher D, Tillich M, Graf A, et |
| 1013 | al. Temporal proteomics of inducible RNAi lines of Clp protease subunits identifies |
| 1014 | putative protease substrates. Plant Physiol. 2018;176(2):1485-508. |
| 1015  1016 | 78. Gou M, Shi Z, Zhu Y, Bao Z, Wang G, Hua J. The F‐ box protein CPR1/CPR30 negatively regulates R protein SNC1 accumulation. Plant J. 2012;69(3):411-20. |
| 1017 | 79. Trujillo M, Shirasu K. Ubiquitination in plant immunity. Current opinion in plant |
| 1018 | biology. 2010;13(4):402-8. Epub 2010/05/18. doi: 10.1016/j.pbi.2010.04.002. |
| 1019 | PubMed PMID: 20471305. |
| 1020 | 80. Suty L, Lequeu J, Lançon A, Etienne P, Petitot A-S, Blein J-P. Preferential |
| 1021 | induction of 20S proteasome subunits during elicitation of plant defence reactions: |
| 1022 | towards the characterization of “plant defence proteasomes”. Int J Biochem Cell B. |
| 1023 | 2003;35(5):637-50. |

| 1024 | 81. Sahu PP, Sharma N, Puranik S, Chakraborty S, Prasad M. Tomato 26S |
| --- | --- |
| 1025 | Proteasome subunit RPT4a regulates ToLCNDV transcription and activates |
| 1026 | hypersensitive response in tomato. Sci Rep. 2016;6(1):1-12. |
| 1027 | 82. Liu J, Pang X, Cheng Y, Yin Y, Zhang Q, Su W, et al. The Hsp70 gene family in |
| 1028 | *Solanum tuberosum*: genome-wide identification, phylogeny, and expression |
| 1029 | patterns. Sci Rep. 2018;8(1):1-11. |
| 1030 | 83. Cha J-Y, Ahn G, Kim JY, Kang SB, Kim MR, Su'udi M, et al. Structural and |
| 1031 | functional differences of cytosolic 90-kDa heat-shock proteins (Hsp90s) in |
| 1032 | *Arabidopsis thaliana*. Plant Physiol Biochem. 2013;70:368-73. |
| 1033 | 84. Song T, Ma Z, Shen D, Li Q, Li W, Su L, et al. An oomycete CRN effector |
| 1034 | reprograms expression of plant HSP genes by targeting their promoters. PLoS |
| 1035 | Pathog. 2015;11(12):e1005348. |
| 1036 | 85. Lee JH, Lee SE, Oh S, Seo E, Choi D. HSP70s Enhance a *Phytophthora infestans* |
| 1037 | Effector-Induced Cell Death via an MAPK Cascade in *Nicotiana benthamiana*. Mol |
| 1038 | Plant Microbe Interact. 2018;31(3):356-62. |
| 1039 | 86. Botër M, Amigues B, Peart J, Breuer C, Kadota Y, Casais C, et al. Structural and |
| 1040 | functional analysis of SGT1 reveals that its interaction with HSP90 is required for |
| 1041 | the accumulation of Rx, an R protein involved in plant immunity. Plant Cell. |
| 1042 | 2007;19(11):3791-804. |
| 1043 | 87. Takahashi A, Casais C, Ichimura K, Shirasu K. HSP90 interacts with RAR1 and |
| 1044 | SGT1 and is essential for RPS2-mediated disease resistance in *Arabidopsis*. Proc |
| 1045 | Natl Acad Sci U S A. 2003;100(20):11777-82. |
| 1046 | 88. Srivastava R, Deng Y, Shah S, Rao AG, Howell SH. BINDING PROTEIN is a |
| 1047 | master regulator of the endoplasmic reticulum stress sensor/transducer bZIP28 in |
| 1048 | *Arabidopsis*. Plant Cell. 2013;25(4):1416-29. |
| 1049 | 89. Reis PA, Rosado GL, Silva LA, Oliveira LC, Oliveira LB, Costa MD, et al. The |
| 1050 | binding protein BiP attenuates stress-induced cell death in soybean via modulation |
| 1051 | of the N-rich protein-mediated signaling pathway. Plant Physiol. 2011;157(4):1853- |
| 1052 | 65. |
| 1053 | 90. Wang D, Weaver ND, Kesarwani M, Dong X. Induction of protein secretory |
| 1054 | pathway is required for systemic acquired resistance. Science. 2005;308(5724):1036- |
| 1055 | 40. |
| 1056 | 91. Ahumada I, Cairó A, Hemmerlin A, González V, Pateraki I, Bach TJ, et al. |
| 1057 | Characterisation of the gene family encoding acetoacetyl-CoA thiolase in |
| 1058 | *Arabidopsis*. Funct Plant Biol. 2008;35(11):1100-11. |
| 1059 | 92. Carrie C, Murcha MW, Millar AH, Smith SM, Whelan J. Nine 3-ketoacyl-CoA |
| 1060 | thiolases (KATs) and acetoacetyl-CoA thiolases (ACATs) encoded by five genes in |
| 1061 | *Arabidopsis thaliana* are targeted either to peroxisomes or cytosol but not to |
| 1062 | mitochondria. Plant Mol Biol. 2007;63(1):97-108. |
| 1063 | 93. Jin H, Song Z, Nikolau BJ. Reverse genetic characterization of two paralogous |
| 1064 | acetoacetyl CoA thiolase genes in *Arabidopsis* reveals their importance in plant |
| 1065 | growth and development. Plant J. 2012;70(6):1015-32. |
| 1066 | 94. Vick BA, Zimmerman DC. Biosynthesis of jasmonic acid by several plant species. |
| 1067 | Plant Physiol. 1984;75(2):458-61. |
| 1068 | 95. Pollmann S, Springer A, Rustgi S, Von Wettstein D, Kang C, Reinbothe C, et al. |
| 1069 | Substrate channeling in oxylipin biosynthesis through a protein complex in the |
| 1070 | plastid envelope of *Arabidopsis thaliana*. J Exp Bot. 2019;70(5):1483-95. |
| 1071 | 96. Tanaka S, Han X, Kahmann R. Microbial effectors target multiple steps in the |
| 1072 | salicylic acid production and signaling pathway. Front Plant Sci. 2015;6:349. |

1073

1074

1075

1076

1077

1078

1079

1080

1081

1082

1083

1084

1085

1086

1087

97. Starý T, Satková P, Piterková J, Mieslerová B, Luhová L, Mikulík J, et al. The elicitin β-cryptogein’s activity in tomato is mediated by jasmonic acid and ethylene signalling pathways independently of elicitin–sterol interactions. Planta. 2019;249(3):739-49.
98. Caplan JL, Kumar AS, Park E, Padmanabhan MS, Hoban K, Modla S, et al. Chloroplast stromules function during innate immunity. Dev Cell. 2015;34(1):45-57.
99. Nejat N, Rookes J, Mantri NL, Cahill DM. Plant–pathogen interactions: toward development of next-generation disease-resistant plants. Crit Rev Biotechnol. 2017;37(2):229-37.
100. Kumar AS, Park E, Nedo A, Alqarni A, Ren L, Hoban K, et al. Stromule extension along microtubules coordinated with actin-mediated anchoring guides perinuclear chloroplast movement during innate immunity. eLife. 2018;7:e23625.

# Supporting information

1088

**S1 Fig. Biological Material.** Information on biological material and procedures

1089

performed in the experimental assay.

1090

**S1 File. Materials and Methods.** Detailed description of LC-MS Materials and

1091

Methods.

1092

##### S1 Table. Proteins identified and quantified in cork oak leaves. List of the 424

1093

proteins identified and quantified in cork oak leaves using the *Arabidopis* proteome

1094

database as reference.

1095

##### S2 Table. Significantly enriched GO Biological Process terms and groups in the list

1096

##### of 80 differential proteins.

1097

##### S3 Table. Significantly enriched GO Cellular Component terms and groups in the

1098

##### list of 80 differential proteins.

1099

##### S4 Table. Significantly enriched GO Biological Process groups in the list of 80

1100

##### differential proteins.

1101

##### S5 Table. Significantly enriched GO Cellular Component groups in the list of 80

1102

##### differential proteins.

Figure 1	[Click here to access/download;Figure;Fig1.tif](https://www.editorialmanager.com/pone/download.aspx?id=27422741&amp;guid=feb6d097-eef6-4f89-9f9f-15c1c85fd002&amp;scheme=1)

Figure 2	[Click here to access/download;Figure;Fig2.tif](https://www.editorialmanager.com/pone/download.aspx?id=27422742&amp;guid=9b94df94-7d0f-4237-b58e-290aad4af426&amp;scheme=1) !;

**Se.v,en1 mOfih afterw .ocumtion**

**Ttme of moculation. wiat *R ciirnm,u:m.,i***

***C***

Figure 3	[Click here to access/download;Figure;Fig3.tif](https://www.editorialmanager.com/pone/download.aspx?id=27422743&amp;guid=799f518e-d133-40a0-8f8f-6b06e5377ac4&amp;scheme=1) !;

***Giles***

*2020-10-19 02:43:04*

--------------------------------------------

In the caption need to indicate what blue and yellow refer to for easy clarity for the reader please.

**FC>2 v <0..5**

**6**

••

**o** *I*

Figure 4	[Click here to access/download;Figure;Fig4.tif](https://www.editorialmanager.com/pone/download.aspx?id=27422745&amp;guid=c295c92e-9823-45d2-a761-f5c30e87b8ce&amp;scheme=1)

***Giles***

*2020-10-19 02:35:10*

--------------------------------------------

Clarity poor in the wording- hard to read. Can resolution be improved?

Figure 5	[Click here to access/download;Figure;Fig5.tif](https://www.editorialmanager.com/pone/download.aspx?id=27422746&amp;guid=1e60f778-116b-4b0b-851a-ec327d7aa0fe&amp;scheme=1)

Figure S1

[Click here to access/download](https://www.editorialmanager.com/pone/download.aspx?id=27422754&amp;guid=eb81fc83-66ae-46d8-bb56-69818f1daf31&amp;scheme=1)

[Supporting Information S1_Fig.tif](https://www.editorialmanager.com/pone/download.aspx?id=27422754&amp;guid=eb81fc83-66ae-46d8-bb56-69818f1daf31&amp;scheme=1)

Document

[Click here to access/download](https://www.editorialmanager.com/pone/download.aspx?id=27422755&amp;guid=2bd8eb17-7f96-431b-8ffe-1547a2d46ae6&amp;scheme=1)

[Supporting Information S1_File.pdf](https://www.editorialmanager.com/pone/download.aspx?id=27422755&amp;guid=2bd8eb17-7f96-431b-8ffe-1547a2d46ae6&amp;scheme=1)

Table S1

[Click here to access/download](https://www.editorialmanager.com/pone/download.aspx?id=27422756&amp;guid=da39466e-6ea1-419c-9f03-a53e0119f8a5&amp;scheme=1)

[Supporting Information S1_Table.pdf](https://www.editorialmanager.com/pone/download.aspx?id=27422756&amp;guid=da39466e-6ea1-419c-9f03-a53e0119f8a5&amp;scheme=1)

Table S2

[Click here to access/download](https://www.editorialmanager.com/pone/download.aspx?id=27422757&amp;guid=08e6db77-1086-456c-a8cb-53739b78ac7b&amp;scheme=1)

[Supporting Information S2_Table.pdf](https://www.editorialmanager.com/pone/download.aspx?id=27422757&amp;guid=08e6db77-1086-456c-a8cb-53739b78ac7b&amp;scheme=1)

Table S3

[Click here to access/download](https://www.editorialmanager.com/pone/download.aspx?id=27422758&amp;guid=163933a2-c25c-4ffe-892d-acbfa62f300e&amp;scheme=1)

[Supporting Information S3_Table.pdf](https://www.editorialmanager.com/pone/download.aspx?id=27422758&amp;guid=163933a2-c25c-4ffe-892d-acbfa62f300e&amp;scheme=1)

Table S4

[Click here to access/download](https://www.editorialmanager.com/pone/download.aspx?id=27422759&amp;guid=575b9b92-22e5-44ad-abad-8c34903f7921&amp;scheme=1)

[Supporting Information S4_Table.pdf](https://www.editorialmanager.com/pone/download.aspx?id=27422759&amp;guid=575b9b92-22e5-44ad-abad-8c34903f7921&amp;scheme=1)

Table S5

[Click here to access/download](https://www.editorialmanager.com/pone/download.aspx?id=27422760&amp;guid=0f9eb815-63a2-4219-9ffd-687cd6b058ad&amp;scheme=1)

[Supporting Information S5_Table.pdf](https://www.editorialmanager.com/pone/download.aspx?id=27422760&amp;guid=0f9eb815-63a2-4219-9ffd-687cd6b058ad&amp;scheme=1)

Data Review URL

[Click here to download Data Review URL http://www.ebi.ac.uk/pride](http://www.ebi.ac.uk/pride)
